# Network pharmacology and molecular docking elucidate potential mechanisms of *Eucommia ulmoides* in hepatic ischemia–reperfusion injury

**DOI:** 10.1038/s41598-023-47918-8

**Published:** 2023-11-24

**Authors:** Xuan Ma, Bochen Pan, Liusong Wang, Zanjie Feng, Cijun Peng

**Affiliations:** 1 Department of General Surgery, Affiliated Hospital of Zunyi Medical University, Zunyi, 563000 Guizhou China; 2 Department of Hepatobiliary Surgery, The affiliated Hospital of Guizhou Medical University, Guiyang, Guizhou China; 3 Clinical Medical Research Center, The affiliated Hospital of Guizhou Medical University, Guiyang, Guizhou China; 4 Department of Biochemistry and Molecular Biology, Zunyi Medical University, Zunyi, 563000, Guizhou China

**Keywords:** Hepatitis, Liver diseases, Molecular biology, Diseases, Molecular medicine

## Abstract

*Eucommia ulmoides* (EU) and its diverse extracts have demonstrated antioxidative, anti-inflammatory, and cytoprotective properties against hepatic ischemia–reperfusion injury (HIRI). However, the primary constituents of EU and their putative mechanisms remain elusive. This study aims to explore the potential mechanisms of EU in the prevention and treatment of HIRI by employing network pharmacology and molecular docking methodologies. The main components and corresponding protein targets of EU were searched in the literature and TCMSP, and the compound target network was constructed by Cytoscape 3.9.1. Liver ischemia–reperfusion injury targets were searched in OMIM and GeneCards databases. The intersection points of compound targets and disease targets were obtained, and the overlapping targets were imported into the STRING database to construct the PPI network. We further analyzed the targets for GO and KEGG enrichment. Finally, molecular docking studies were performed on the core targets and active compounds. The component-target network unveiled a total of 26 efficacious bioactive compounds corresponding to 207 target proteins. Notably, the top-ranking compounds based on degree centrality were quercetin, β-sitosterol, and gallic acid. Within the PPI network, the highest degree centrality encompassed RELA, AKT1, TP53. GO and KEGG enrichment analysis elucidated that EU in HIRI primarily engaged in positive regulation of gene expression, positive transcriptional regulation via RNA polymerase II promoter, negative modulation of apoptotic processes, positive regulation of transcription from DNA templates, and drug responsiveness, among other biological processes. Key pathways included cancer pathways, RAGE signaling pathway, lipid metabolism, atherosclerosis, TNF signaling pathway, PI3K-Akt signaling pathway, and apoptotic pathways. Molecular docking analysis revealed robust affinities between quercetin, β-sitosterol, gallic acid, and RELA, AKT1, TP53, respectively. This study reveals EU exhibits substantial potential in mitigating and treating HIRI through multifaceted targeting and involvement in intricate signaling pathways.

## Introduction

Hepatic ischemia–reperfusion injury (HIRI) refers to the pathological phenomenon where the liver undergoes additional damage upon the restoration of blood flow after a period of ischemia, during which liver cells experience a transient ischemic environment^1,2^. It is a common clinical issue in diseases such as liver transplantation, liver surgery, shock, and trauma^3^. End-stage liver disease is a significant contributor to increased mortality rates associated with liver diseases worldwide, and liver transplantation remains the optimal treatment method for various end-stage liver diseases^4^. Despite the continuous development and replacement of precise minimally invasive hepatobiliary surgery and surgical techniques, HIRI remains a major cause of postoperative liver dysfunction and even multi-organ failure^5,6^. The occurrence of HIRI during surgery significantly increases the risk of acute and chronic rejection reactions in liver transplantation, substantially affecting the success rate of transplantation, exacerbating the shortage of available livers, and being closely related to early graft dysfunction^3,7,8^. Therefore, preventing and mitigating HIRI during surgery has been a persistent concern for hepatobiliary surgeons, and pharmacological preconditioning and targeted gene interventions have emerged as key research topics in both domestic and international HIRI prevention and treatment studies. Research has demonstrated that energy metabolism regulators, antioxidants, adenosine receptor agonists, calcium channel blockers, and other agents exhibit protective effects against HIRI^9,10^. Although there are literature reports indicating that pre-operative and post-operative pharmacological interventions can alleviate HIRI, such approaches have proven effective primarily in patients with limited liver tissue resection and prolonged ischemic time^11^. Moreover, many of the aforementioned drugs have substantial toxic side effects, hindering their development and clinical application. To date, there has been limited breakthrough progress in the prevention and treatment of HIRI. Therefore, the prevention and treatment of HIRI necessitate the exploration of safer and more effective therapeutic strategies.

Traditional Chinese medicine (TCM) has been widely used in China for thousands of years to treat common and complex diseases, and its therapeutic efficacy has been well recognized throughout the long history of medicinal practices in China. TCM possesses advantages such as multiple targets, a wide safety range, and minimal toxicity and side effects. Research has found that TCM exhibits protective effects against HIRI, making the development of TCM monomers and herbal formulations with protective effects against HIRI a promising research direction in the near future. Interestingly, previous studies have shown that preoperative treatment with extracts of *Eucommia ulmoides* (EU), Salvia miltiorrhiza (SM), and resveratrol have clear preventive and therapeutic effects against HIRI. EU, a representative kidney-tonifying herb in China, is described in the classic TCM text "Ben Cao Gang Mu" as a liver-regulating herb that nourishes the liver, moistens dryness, tonifies liver deficiency, and possesses medicinal properties that were previously undiscovered. EU has a purple color and a sweet and slightly spicy taste, with a neutral and warm energy. It can enter the liver to tonify the kidneys. EU contains numerous active components, including phenylethanoid glycosides, lignans, terpenoids, polysaccharides, and flavonoids, which have pharmacological effects such as anti-tumor, anti-inflammatory, disinfectant, hepatoprotective, choleretic, and antioxidant properties. Additionally, EU can effectively treat conditions such as lumbago and knee pain, weak fetal retention, and scrotal itching caused by liver and kidney deficiency^12,13^. In recent years, there have been literature reports indicating that EU and its chemical constituents have protective effects against liver fibrosis, fatty liver, HIRI, and liver damage caused by carbon tetrachloride^14–18^. However, in our early studies on rats, we discovered that EU and some of its monomer components (such as chlorogenic acid, aucubin, and catalpol) could alleviate HIRI when administered as pretreatment to the rats. The mechanism behind this effect involves reducing the generation of reactive oxygen species (ROS) during HIRI and inhibiting the sterile inflammatory response mediated by HMGB1. It is evident that the role of EU in HIRI is worth acknowledging^19,20^.

Due to the complex composition of TCM, with multiple targets and a wide range of therapeutic signaling pathways, in-depth research on traditional Chinese medicine poses challenges that cannot be addressed by traditional research methods alone, as they cannot accurately elucidate the molecular mechanisms of drug actions^21^. Network pharmacology and molecular docking are powerful computational methods that can reveal the interactions between drugs and biological systems^22–24^. Network pharmacology helps uncover the complex interactions between multiple components of a drug and the corresponding target pathways in disease^25^. On the other hand, molecular docking can predict the binding affinity and structural interactions between active compounds in EU and protein targets related to HIRI^26^. The integration of network pharmacology and molecular docking can provide a comprehensive understanding of the potential mechanisms of EU in HIRI^27^. By identifying key targets and pathways, this approach can help elucidate the molecular basis of EU’s therapeutic effects and guide the development of new treatment strategies for HIRI.

In this study, our aim is to explore the potential mechanisms of EU in HIRI using network pharmacology and molecular docking techniques. We will identify the active compounds in EU, predict their targets, and construct a drug-target interaction network. Additionally, we will use molecular docking simulations to validate the predicted targets and assess the binding affinity and stability of compound-target interactions. Understanding the molecular mechanisms of EU in HIRI can help in the development of new therapies and target mechanisms for the prevention and treatment of this severe disease. It provides a more favorable theoretical basis for the clinical application of EU in intervening HIRI. Furthermore, this research can provide scientific evidence for traditional medicine and promote the integration of traditional Chinese medicine with modern medicine in the field of liver diseases.

## Materials and methods

### Screening of active components and related targets of *Eucommia ulmoides*

The active components of EU were collected using the Traditional Chinese Medicine Systems Pharmacology Database (TCMSP, https://old.tcmsp-e.com/tcmsp.php)^28^ and relevant literature review. Filtering criteria including oral bioavailability (OB) ≥ 30% and drug-likeness (DL) ≥ 0.18 were applied to obtain active compounds. The related targets of these active compounds were retrieved from the "Related Targets" section. For compounds without target information, the Canonical SMILES of the active substances were obtained from the PubChem database (https://pubchem.ncbi.nlm.nih.gov/)^29^ using their CAS numbers. The obtained Canonical SMILES were then input into the Swiss Target Prediction database (http://www.swisstargetprediction.ch/) to retrieve target information. The UniProt database (https://www.uniprot.org/)^30^ was further utilized to obtain gene symbols and IDs for the target proteins.

### Construction of the active compound-target network

Cytoscape^31^, a network biology visualization and analysis application, was employed to visualize and construct a network of active compounds and their corresponding targets. The potential active components and matched targets of EU were imported into Cytoscape 3.9.1. Each component or target was represented by a node, and the relationships between components and targets were represented by connecting lines.

### Determination of HIRI prediction targets

Information on the disease targets related to hepatic ischemia–reperfusion injury (HIRI) was collected from the Online Mendelian Inheritance in Man (OMIM) database (https://www.omim.org/)^32^ and GeneCards database (https://www.genecards.org/)^33^. A search using the keyword “Hepatic ischemia–reperfusion injury” was performed to obtain HIRI-related targets from each database. The target results from both databases were merged and duplicates were removed. The remaining genes were collected and used to investigate the information of HIRI disease target genes. Venn analysis of the potential targets of EU and HIRI was performed using an online website (www.bioinformatics.com.cn) to visualize the intersection.

### Construction of the protein–protein interaction (PPI) network and selection of core targets

The overlapping targets of EU and HIRI were imported into the STRING database (https://cn.string-db.org/)^34^ for PPI analysis. The analysis was performed using the “Homo sapiens” organism and a minimum required interaction score of 0.900. The resulting information on protein–protein interactions was used to construct a protein–protein interaction network. The exported node–node data was visualized and analyzed using Cytoscape 3.9.1. The network analysis plugin was employed to analyze the network's degree centrality (DC), betweenness centrality (BC), and closeness centrality (CC) to investigate the network's topological properties.

### GO enrichment and KEGG pathway analysis

To study the biological functions of potential targets in the anti-HIRI effects of EU, the Gene Ontology (GO) analysis and KEGG pathway data were collected using the Database for Annotation^35^, Visualization, and Integrated Discovery (DAVID, https://david.ncifcrf.gov/). GO analysis was used to identify biological processes (BP), cellular components (CC), and molecular functions (MF). KEGG pathway analysis helped identify important signaling pathways involved in biological processes. The GO and KEGG data were uploaded to the bioinformatics platform (http://www.bioinformatics.com.cn/) for visualization and analysis.

### Molecular docking

Molecular docking is a commonly used method in drug discovery to study the recognition and interaction between receptors and ligands^36^. It is a theoretical simulation method that combines modeling and affinity to investigate molecular interactions and predict binding modes. AutoDock 4 (1.5.6) was used to perform molecular docking of the active components of EU with the key targets, predicting their binding modes and binding affinities. The following process was conducted:

- The active components downloaded from the Protein Data Bank (PDB) were saved in mol2 format and imported into Chembio3D software for energy minimization. AutodockTools-1.5.6 software was used to add hydrogens, calculate charges, and assign charges. The ligand flexibility was set in the Ligand option and saved as a “pdbqt” format file.
- The key target proteins were downloaded from the PDB (http://www.rcsb.org/) website. The proteins were imported into PyMOL (2.3.0) software to extract the original ligands and remove water molecules. The proteins were then imported into AutoDockTools (v1.5.6) software to add hydrogens, calculate charges, and assign charges. The atom types were set as Macromolecule and saved as a "pdbqt" format file.
- In AutoDockTools (v1.5.6) software, the grid box was centered on the original ligand of the protein or around the reported key amino acid residues if no suitable original ligand was available.
- The docking box size was set to 80 × 80 × 80 (grid spacing of 0.375 Å), and other parameters were kept at their default values.
- The results were analyzed and processed using PyMOL and Ligplot software.

## Results

### Screening of effective components of *Eucommia ulmoides* and investigation of HIRI-related targets

**Figure 1:**
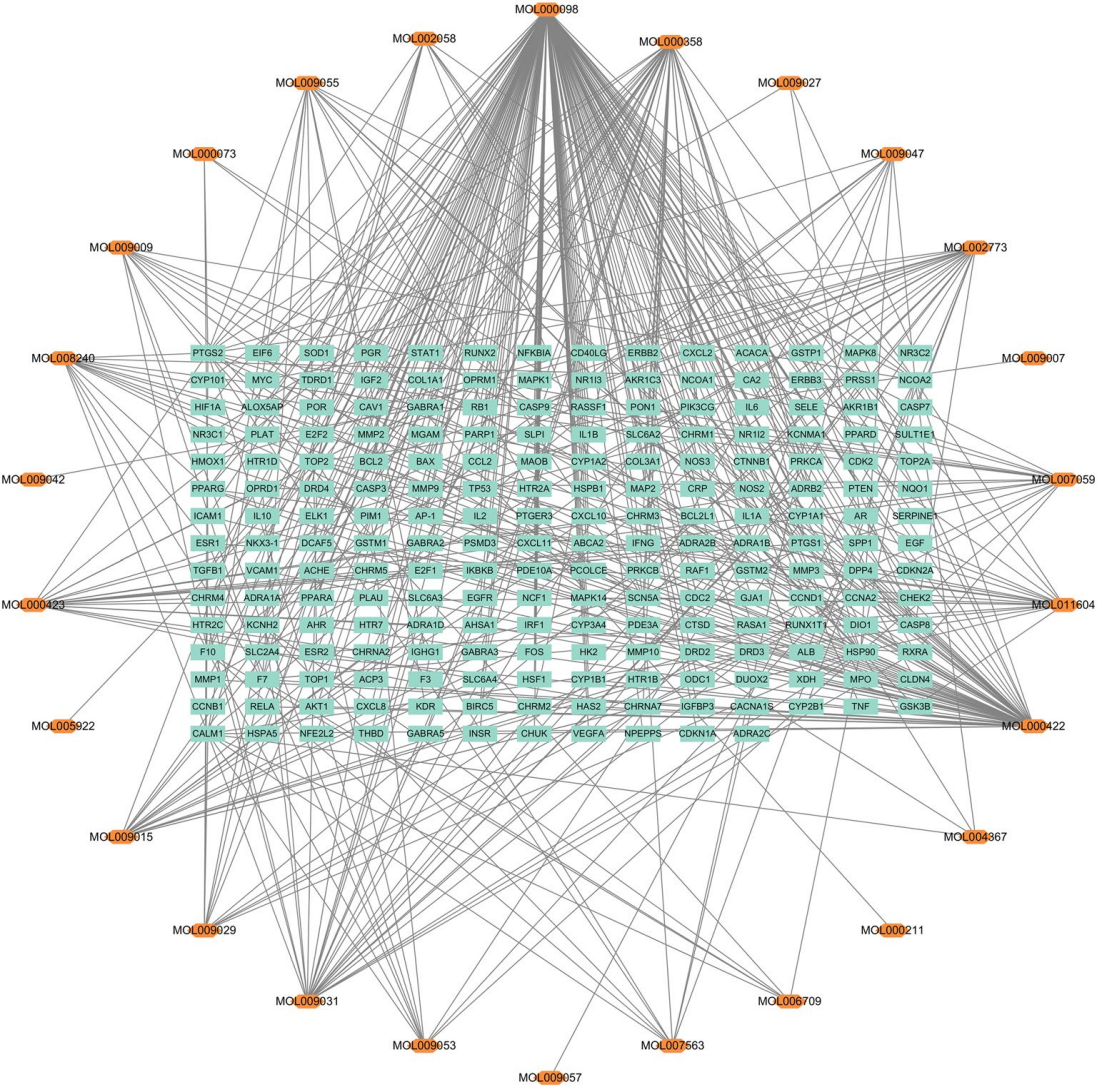
The ingredient-target network analysis. The orange hexagonal nodes represent the main active ingredients of EU, and the Cyan rectangle represent the potential targets of EU for treating HIRI. EU = *Eucommia ulmoides* cortex, HIRI = hepatic ischemia–reperfusion injury.

**Figure 2:**
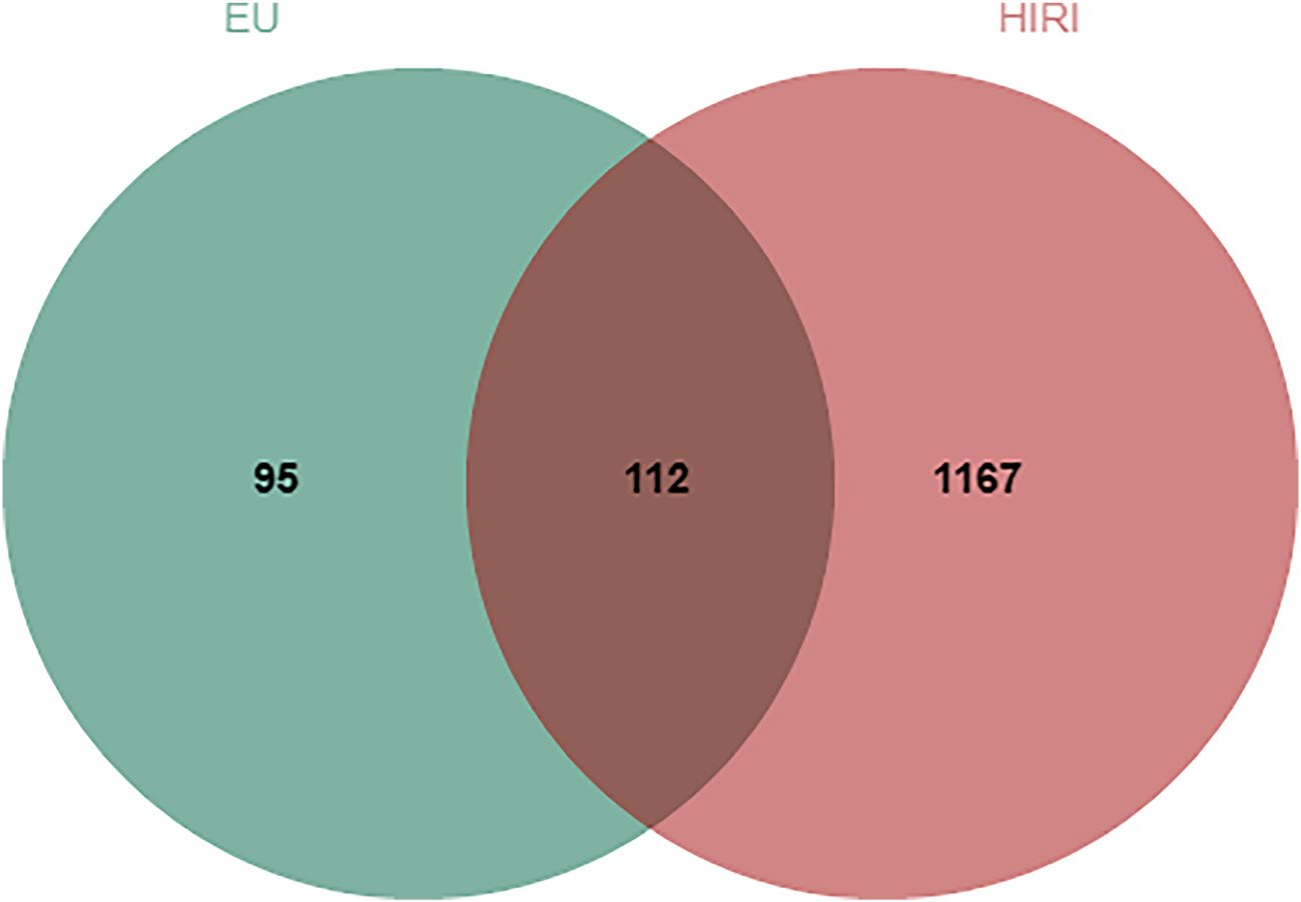
The Venn diagram of the targets both in EU and HIRI. The overlap part indicates the potential therapeutic targets by which EU treats HIRI. EU = *Eucommia ulmoides* cortex, HIRI = hepatic ischemia–reperfusion injury.

A search for the keywords “*Eucommia ulmoides*” or “*Eucommia ulmoides* bark” in the TCMSP database resulted in 147 *Eucommia ulmoides*-related active components. After filtering based on drug-likeness (DL) ≥ 0.18 and oral bioavailability (OB) ≥ 30%, 26 effective active components were selected. By analyzing the “Related Targets” information of these active components, a total of 479 target proteins were obtained. After removing duplicates, 207 target proteins were found to be associated with EU. Given the diverse active components and multiple target interactions of EU, we constructed a network of EU components and targets, as shown in Fig. 1. The central green square represents the potential target proteins of EU, surrounded by the effective active components, resulting in a network with 233 nodes and 479 edges. Table 1 presents the top 10 entries selected based on their degree values. A search in the OMIM database and GeneCards database yielded a total of 1279 target proteins related to HIRI. The overlapping target genes between the target proteins of EU and HIRI were visualized using the Venn diagram on the Venny online analysis website. A total of 112 overlapping genes were identified and presented in the Venn diagram shown in Fig. 2. These overlapping genes are considered potential therapeutic targets through which EU exerts its preventive and therapeutic effects against HIRI.

**Table 1:** Top 10 compounds information network of EU.

Mol ID	Molecule structure	Compound	Degree	OB	DL
MOL000098		Quercetin	149	46.43	0.28
MOL000422		Kaempferol	59	41.88	0.24
MOL000358		Beta-sitosterol	37	36.91	0.75
MOL009031		Cinchonan-9-al, 6'-methoxy-, (9R)-	25	68.22	0.40
MOL002773		Beta-carotene	22	37.18	0.58
MOL000443		Erythraline	20	49.18	0.55
MOL011604		Syringetin	19	36.82	0.37
MOL009015		Tabernemontanine	17	58.67	0.61
MOL007059		3-beta-Hydroxymethyllenetanshiquinone	16	32.16	0.41
MOL009055		hirsutin_qt	15	49.81	0.37

### Construction of PPI network and screening of key genes

**Figure 3:**
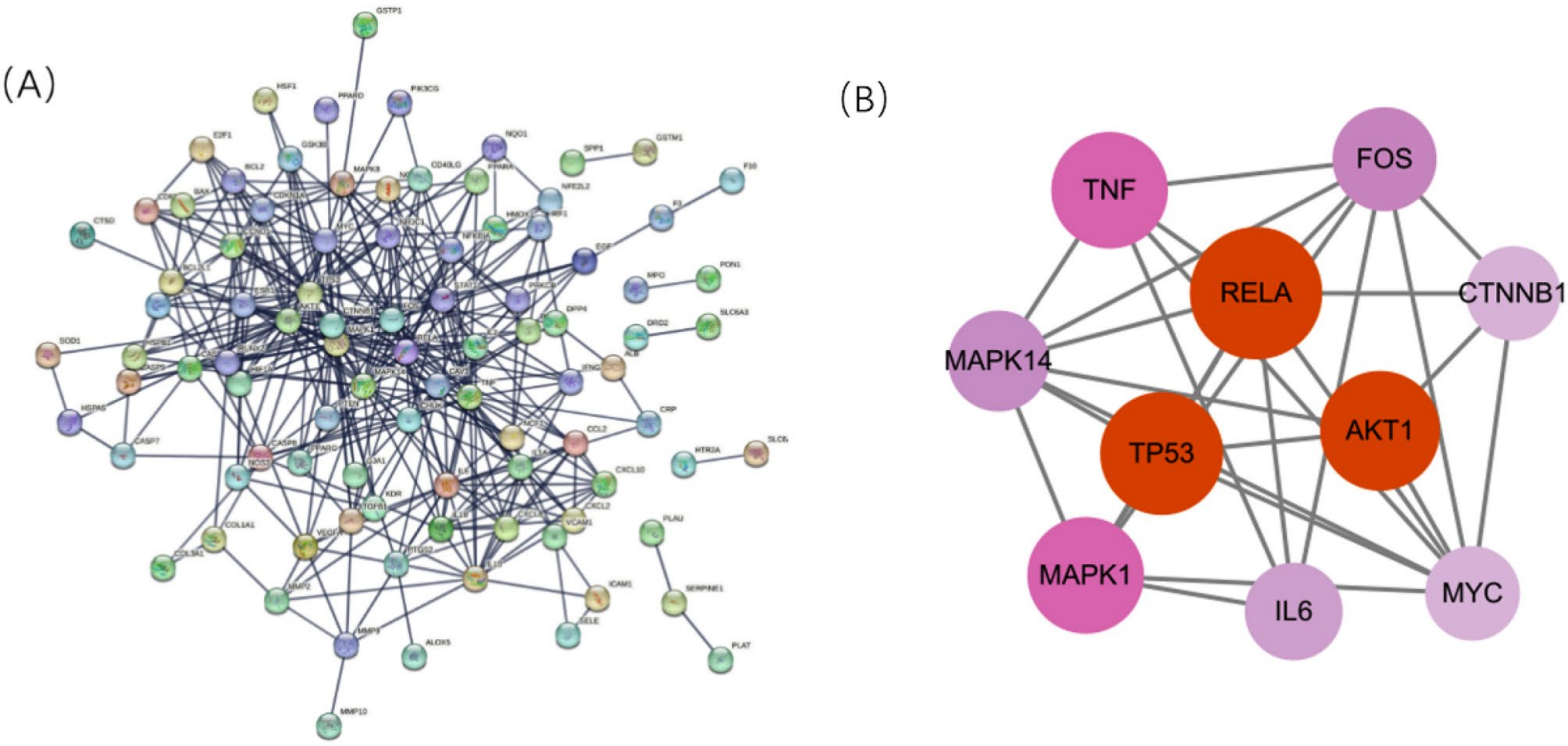
The PPI network analysis of the intersection targets. (**A**) The PPI network was constructed based on the STRING database. (**B**) The top 10 targets with the highest degree value were screened and considered as key targets. PPI = protein–protein interaction, STRING = Search Tool for the Retrieval of Interacting Genes/Proteins.

Based on the Venn diagram results, there were 112 overlapping genes between the potential targets of EU and the target genes associated with HIRI. These common genes were imported into the STRING database to construct the protein–protein interaction (PPI) network. After removing disconnected nodes, the protein interaction network was obtained, as shown in Fig. 3A. The PPI network consisted of 112 protein nodes and 371 interaction edges. The network was saved in TSV format and imported into Cytoscape software for topological analysis. The CentiScaPe plugin was used to identify core genes based on their degree centrality values, and the top 10 core genes were selected to construct the protein–protein interaction network, as shown in Fig. 3B. The nodes in the network were represented by varying sizes and colors, ranging from purple to red. The darker the color and the larger the node size, the higher the probability of being a core target. Detailed information of the top 10 important targets is presented in Table 2.

**Table 2:** PPI network top 10 target information.

Target	Degree	Betweenness centrality	Closeness centrality
RELA	31	0.09884516	0.57446809
TP53	28	0.107575	0.54362416
AKT1	27	0.12150928	0.53642384
MAPK1	26	0.08883322	0.5472973
TNF	26	0.09832991	0.53642384
FOS	22	0.06627332	0.53289474
MAPK14	21	0.05132374	0.52941176
IL6	20	0.05274622	0.5
CTNNB1	19	0.06773229	0.52258065
MYC	19	0.02628284	0.51265823

### GO enrichment and KEGG pathway analysis

**Figure 4:**
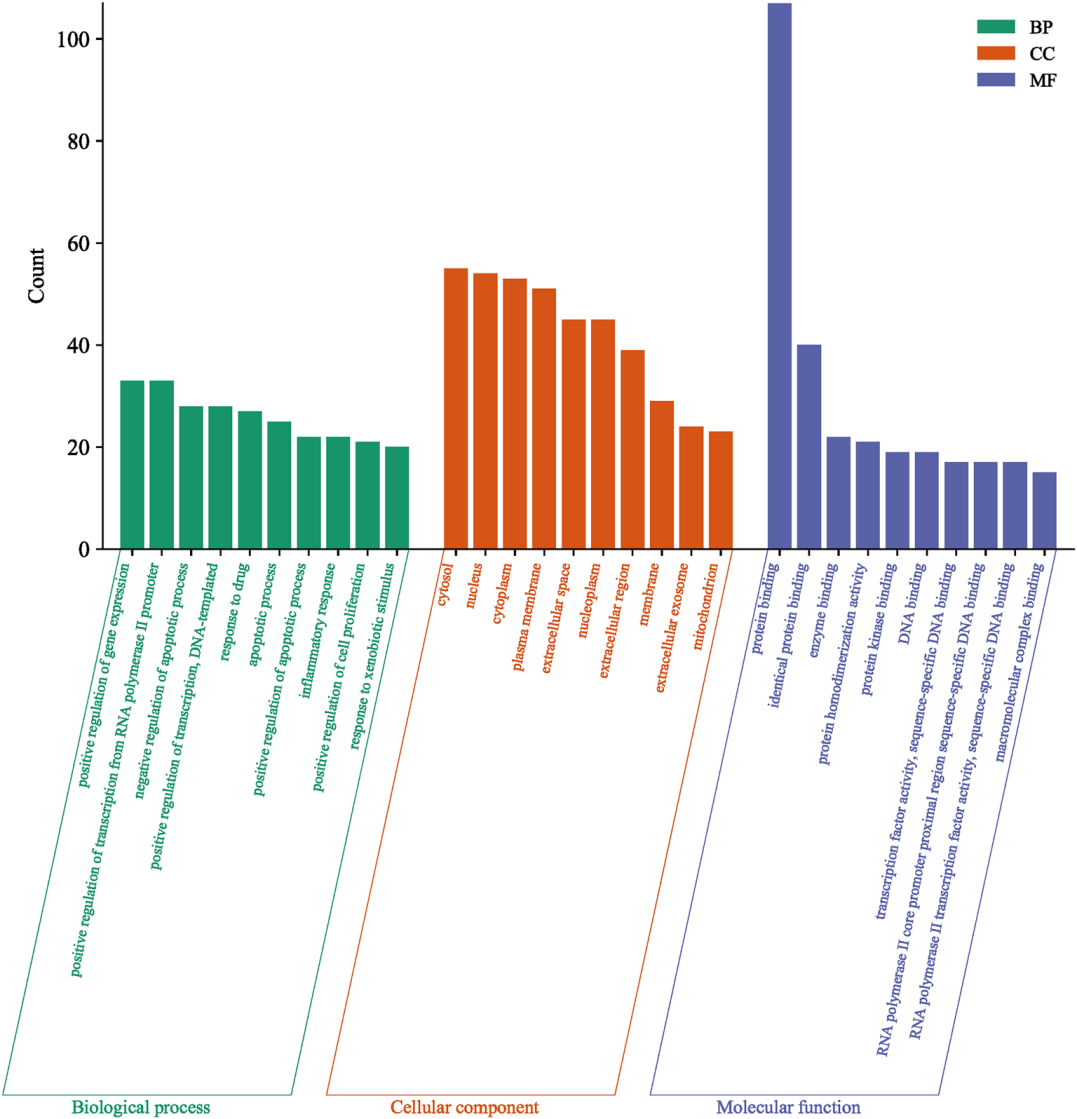
Top 10 GO terms of hub genes.

**Figure 5:**
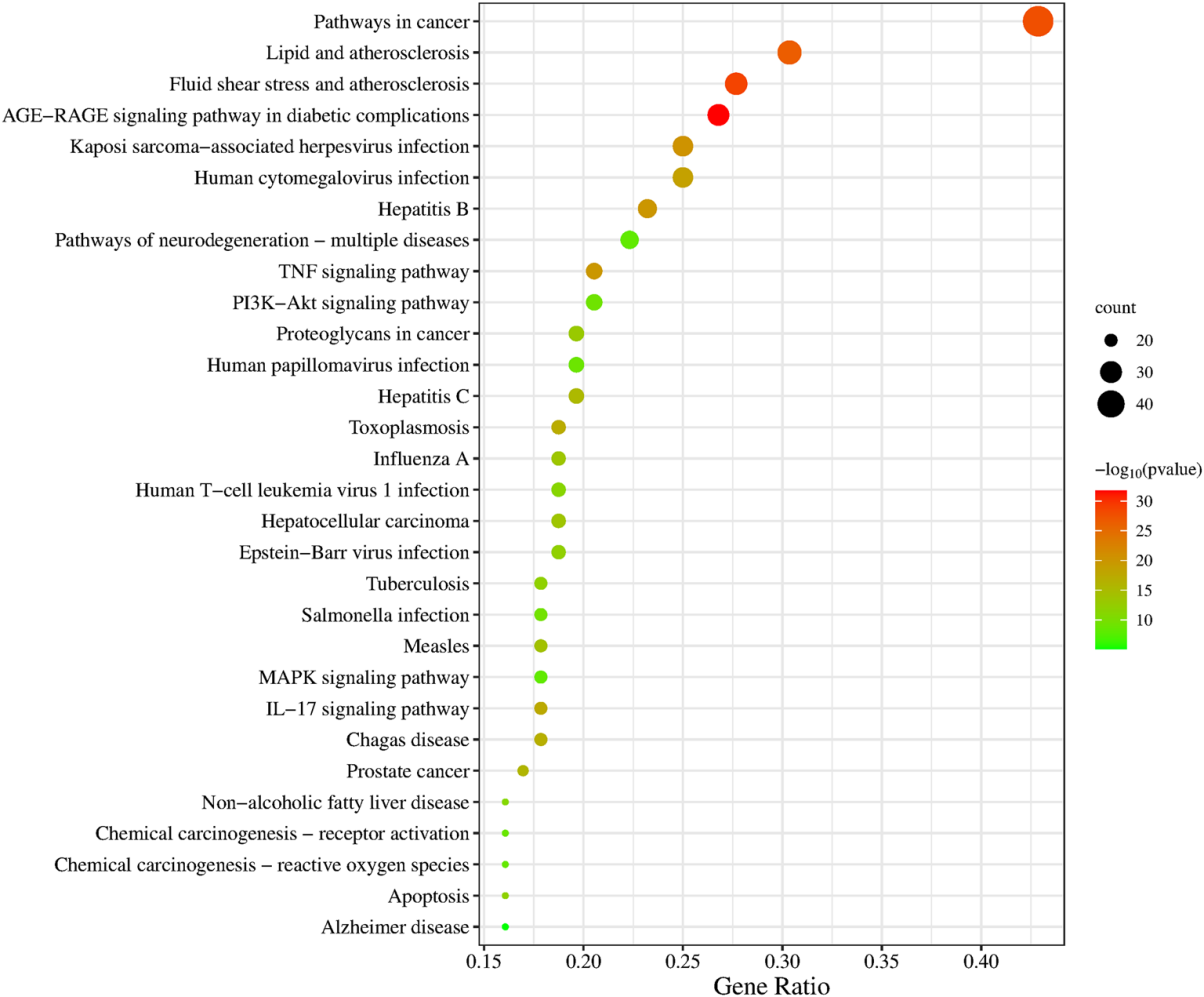
Top 30 KEGG pathways of hub genes.

The overlapping 112 target genes of EU and HIRI were subjected to Gene Ontology (GO) enrichment analysis using the DAVID database. A total of 712 biological processes, 65 cellular components, and 113 molecular functions were obtained under the GO terms. The enrichment analysis was performed with a significance threshold of *p*-value < 0.05, and the results were sorted in descending order based on the count of hit genes. The top 10 enriched terms are shown in Fig. 4. In terms of biological processes, the EU targets associated with HIRI were mainly enriched in positive regulation of gene expression, positive regulation of transcription from RNA polymerase II promoter, negative regulation of apoptotic process, positive regulation of transcription, DNA-templated, and response to drug. In the cellular component category, cytosol, nucleus, cytoplasm, and plasma membrane were enriched. In the molecular function category, the enriched processes mainly included protein binding, identical protein binding, and enzyme binding. To further explore the signaling pathway mechanisms involved in EU’s protection against HIRI, KEGG pathway enrichment analysis was conducted. A total of 162 pathways were enriched. Figure 5 displays the top 30 pathways ranked by the number of enriched genes in each pathway. The enriched pathways were mainly associated with cancer, the AGE-RAGE signaling pathway in diabetic complications, lipid and atherosclerosis, TNF signaling pathway, PI3K-Akt signaling pathway, and apoptosis.

### Molecular docking study

**Figure 6:**
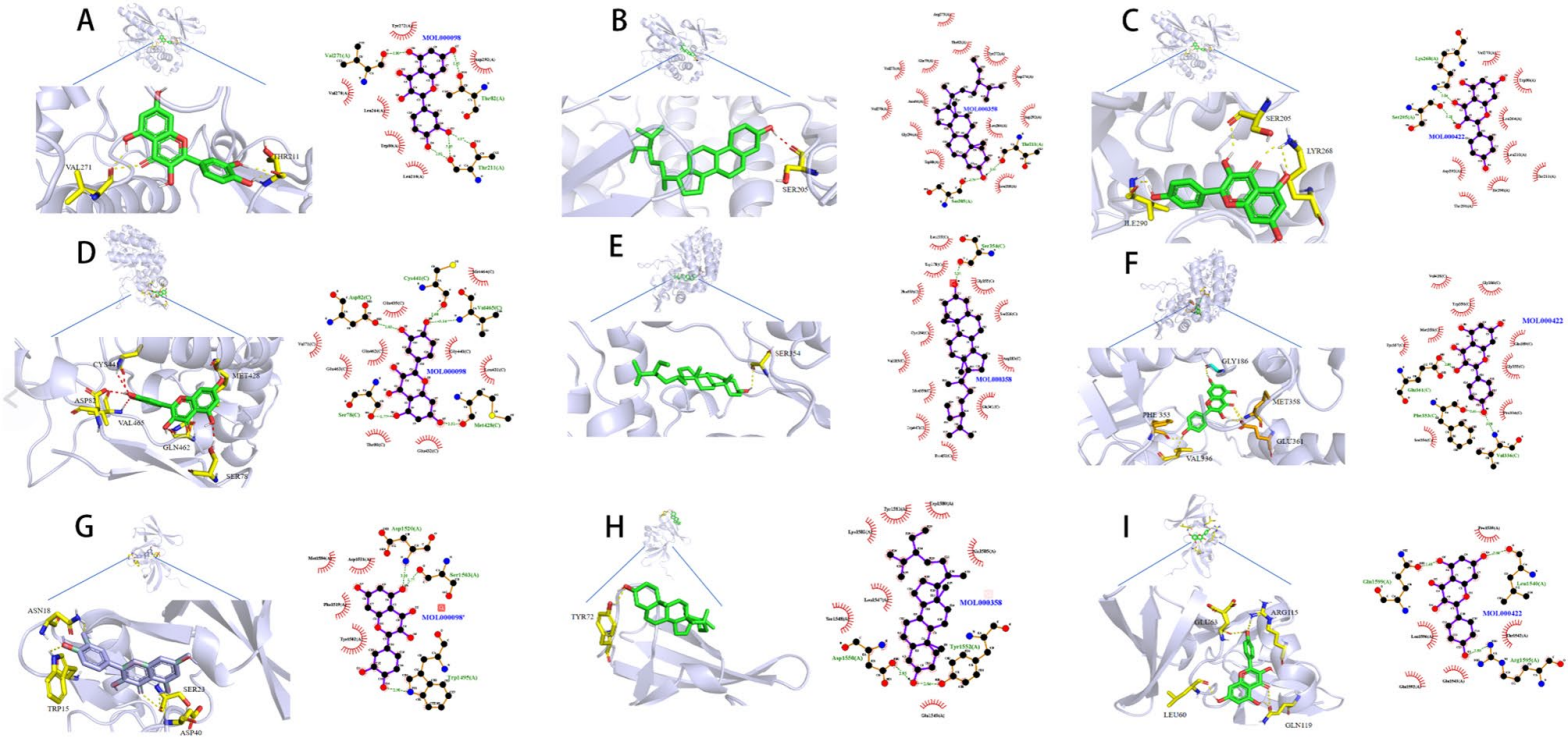
Molecular docking results of main chemical components of EU. (**A**) Quercetin-RELA; (**B**) Quercetin-ATK1; (**C**) Quercetin-TP53; (**D**) Kaempferol- RELA; (**E**) Kaempferol- ATK1; (**F**) Kaempferol-TP53; (**G**) beta-sitosterol-RELA; (**H**) beta-sitosterol- ATK1; (**I**) beta-sitosterol-TP53.

Based on the previous PPI analysis and compound-target network analysis, we selected the top three active compounds: quercetin (MOL000098), paeonol (MOL000422), and β-sitosterol (MOL000358), along with three key target proteins RELA (PDB ID: 1NFI), AKT1 (PDB ID: 3O96), and TP53 (PDB ID: 6MXY). The molecular docking was performed to predict the binding affinity between the active compounds and the target proteins. The docking scores are shown in Table 3. Generally, a binding energy less than 0 indicates a favorable binding between the ligand and the receptor, and a binding energy less than − 5 kcal/mol suggests a strong affinity. Figure 6 visualizes the optimal docking poses between the receptor and the ligands.

**Table 3:** Binding energy of the compound to the core target (kcal/mol).

Target	Target structure	Compound	Affinity (kcal/mol)
RELA		Quercetin	− 6.74
		Kaempferol	− 6.81
		beta-sitosterol	− 5.88
ATK1		Quercetin	− 10.77
		Kaempferol	− 9.65
		beta-sitosterol	− 7.61
TP53		Quercetin	− 7.01
		Kaempferol	− 7.21
		beta-sitosterol	− 6.25

The molecular docking results revealed the interactions between quercetin and RELA, AKT1, and TP53 (Fig. 6A–C). In Fig. 6A, quercetin formed hydrogen bonds with Thr82 and Val271 in RELA. In Fig. 6B, quercetin formed a hydrogen bond with Asp82, Cys441, Val465, Met428, and Ser78 in AKT1. Figure 6C showed the hydrogen bonds between quercetin and Asp1520, Cys1503, and Trp1495 in TP53.

Paeonol was found to interact with RELA, AKT1, and TP53 (Fig. 6D–F). In Fig. 6D, paeonol formed a hydrogen bond with Thr213 and Ser205 in RELA. In Fig. 6E, paeonol interacted with Ser354 in AKT1 through a hydrogen bond. Figure 6F demonstrated the hydrogen bonds between paeonol and Asp1550, Tyr1552, and Thr327 in TP53.

Similarly, β-sitosterol showed interactions with RELA, AKT1, and TP53 (Fig. 6G–I). In Fig. 6G, β-sitosterol formed a hydrogen bond with Ser205 and Lys268 in RELA. In Fig. 6H, β-sitosterol interacted with Phe353, Val336, and Glu361 in AKT1 through a hydrogen bond. Figure 6I illustrated the hydrogen bonds between β-sitosterol and Arg1595, Leu1540, and Glu1599 in TP53.

The lower the binding energy, the higher the affinity and stability of the ligand-receptor complex. The docking results indicated that all of these compounds had binding energies below − 5 kcal/mol, suggesting strong affinities with the target proteins. Among them, the docking between paeonol and RELA showed the lowest binding energy (− 9.65 kcal/mol), while the docking between paeonol and AKT1 exhibited the lowest binding energy (− 10.22 kcal/mol). The docking between paeonol and TP53 had the lowest binding energy (− 7.61 kcal/mol). These three compounds (quercetin, paeonol, and β-sitosterol) demonstrated good binding with the three core targets (RELA, AKT1, and TP53). Based on these findings, we predict that these compounds may play a key role in the treatment of HIRI.

## Discussion

Hepatic ischemia–reperfusion injury (HIRI) is a common complication observed during liver surgeries (such as liver transplantation and hepatectomy) and in the treatment of ischemic liver diseases (such as hepatic artery occlusion and liver infarction). HIRI is also a significant factor that impacts liver function and patient survival following liver transplantation. It gives rise to severe pathological and physiological alterations in the liver, including impaired liver function, hepatocyte apoptosis, inflammatory response, and cellular necrosis^37,38^. In severe cases, it can lead to liver failure and even mortality. Unfortunately, currently, there are no approved pharmaceutical treatments or interventions available for the prevention and management of HIRI. Previous investigations have indicated that calcium channel blockers, adenosine receptor agonists, energy metabolism regulators, and antioxidants demonstrate protective effects against HIRI^9,10^. However, the considerable adverse effects associated with these medications have limited their research to animal models, lacking clinical evidence, and impeding their application in clinical practice. Research on pharmacological pre-conditioning for the prevention of HIRI has yet to achieve significant breakthroughs. Therefore, it is of paramount importance to explore effective and innovative approaches for the prevention and management of HIRI. Traditional Chinese medicine (TCM) has garnered considerable attention in the scholarly community in recent years due to its wide safety range, multitarget effects, and minimal adverse effects^39^.

HIRI is an inevitable consequence of major liver surgery, and the sterile inflammatory response that endangers organ vitality in the early stage of reperfusion is a very important phase of HIRI^40^. We noticed the anti-inflammatory pharmacological effects of EU in our effort to solve the problem of HIRI^41^, and we thought about the pathogenesis of sterile inflammation in HIRI and the anti-inflammatory effects of EU. Interestingly, we found that pre-treating rats with EU and some of its monomer components (chlorogenic acid, aucubin and catalpol) could significantly reduce HIRI, and the mechanism involved reducing the generation of ROS during HIRI and inhibiting the sterile inflammatory response mediated by HMGB1 during HIRI^19,20,42^, thereby exerting a protective effect against HIRI. It can be seen that the role of EU in HIRI is worth affirming. However, the composition of EU is complex^21^, and there are many potential targets and mechanisms of action, many of which are still unknown. Traditional research methods cannot quickly and accurately elucidate the effective active ingredients and specific molecular mechanisms of drug action, so in-depth research faces some challenges. Therefore, it is very important to use computer algorithms such as network analysis and molecular docking to comprehensively strengthen the research on the prevention and treatment of HIRI with EU and its mechanisms. Exploring the protective effect and mechanism of EU on HIRI is an important supplement to drug pretreatment for the prevention and treatment of HIRI, and also lays a theoretical foundation for improving the prognosis of liver resection patients and promoting the development of liver transplantation.

Although several studies have demonstrated the anti-inflammatory effects of EU in hepatic ischemia–reperfusion injury (HIRI), the underlying molecular mechanisms of EU in preventing HIRI remain largely unclear. The aim of this study was to systematically investigate the potential mechanisms of EU in the prevention and treatment of HIRI. Initially, a total of 26 active components of EU, including quercetin, β-sitosterol, and kaempferol, were collected from the TCMSP database and relevant literature. In the component-target network, quercetin exhibited the highest degree, indicating its extensive connectivity. β-sitosterol ranked second in terms of, followed by kaempferol. Quercetin, the most abundant active component in EU, is considered an antioxidant and HO-1 inducer. Its strong HO-1 induction ability contributes to its prominent role in preventing HIRI^43^. Previous studies have also demonstrated that quercetin alleviates HIRI by inhibiting ERK/NF-κB pathway-induced apoptosis and autophagy^44^. Furthermore, in a rat model of HIRI, quercetin liposomes were found to be a valuable tool for addressing the current limitations in HIRI treatment^45^. However, it should be noted that the dosage of quercetin must be carefully considered, as different dosages can lead to varied outcomes^46^. Regarding kaempferol, a previous study on HIRI models suggested that its protective effects are mediated through the activation of the Nrf2/HO-1 signaling pathway, leading to the inhibition of oxidative stress and inflammation^47^. Additionally, kaempferol has demonstrated beneficial effects in liver, brain, lung, kidney, and myocardial ischemia–reperfusion injuries, primarily by suppressing oxidative stress and sterile inflammation^48–53^. Although no specific reports regarding the effects of β-sitosterol on HIRI were found, β-sitosterol has been shown to prevent myocardial ischemia–reperfusion injury (MIRI) both in vivo and in vitro, potentially through the regulation of the PPARγ/NF-κB signaling pathway^54^. Another study demonstrated that β-sitosterol pretreatment increased glutathione activity and superoxide dismutase levels while reducing malondialdehyde expression in the heart and kidneys, thus exerting protective effects against MIRI and renal ischemia–reperfusion injury (KIRI) by limiting inflammatory responses and oxidative stress^55^. Furthermore, β-sitosterol pretreatment reduced ischemic brain injury in rats after unilateral common carotid artery occlusion and exhibited therapeutic effects on damaged blood vessels^56,57^. These findings suggest the significant importance of these active components (quercetin, kaempferol, and β-sitosterol) in the prevention and treatment of HIRI using EU, warranting further exploration.

We collected HIRI disease targets from databases and found that EU shares 112 common targets with HIRI. These target genes are considered potential targets for EU in the prevention and treatment of HIRI. To further identify core target genes from these numerous targets, we constructed a protein–protein interaction (PPI) network. The results revealed that RELA, AKT1, TP53, MAPK1, TNF, MAPK14, FOS, CTNNB1, MYC, and IL6 may be potential core target genes, especially RELA, AKT1, and TP53.

RELA, also known as NF-κB/p65, is one of the five components of the NF-κB transcription factor family. Studies have shown that HIRI significantly induces phosphorylation of NF-κB/p65 and increases the expression of pro-inflammatory cytokines, including IL-6 and TNF-α. However, pretreatment with kaempferol can dose-dependently reverse these effects, thereby alleviating HIRI^47,58^. Recent research has also found that inhibiting NF-κB signaling can reduce inflammation, liver injury, cell apoptosis, and oxidative stress in HIRI mice^58^. Interestingly, in other studies investigating the intervention of HIRI with other herbal monomers, such as chlorogenic acid (CGA) and resveratrol, it was found that they can prevent HIRI by inhibiting TLR-4/NF-κB signaling pathway-mediated inflammation and mitochondria-mediated cell apoptosis^59,60^. TNF-α and IL-6, as typical inflammatory factors and key mediators of HIRI, were also found among our predicted potential targets, along with RELA, TNF, and IL-6. Therefore, it can be inferred that the prevention of HIRI by herbal monomers may primarily occur through the inhibition of RELA (NF-κB/p65) phosphorylation, thereby suppressing the expression of inflammatory factors, oxidative stress reactions, and cell apoptosis.

Akt, a serine/threonine kinase, is an oncogene that has attracted significant attention in the medical field due to its important role in regulating cell growth, metabolism, transcription, proliferation, protein synthesis, and survival. Among the three Akt isoforms (Akt1, Akt2, and Akt3) predominantly expressed in mammals, Akt1 exhibits widespread expression in tissues. Studies by Gao et al. have shown that limb ischemic preconditioning (LIPOC), acidic microenvironment, microRNA-218-5p, and microRNA-494 can alleviate HIRI by modulating the PI3K/Akt signaling pathway, which involves reducing cell apoptosis, inflammatory responses, and oxidative stress^61–65^.

TP53 is a crucial tumor suppressor gene. Interestingly, previous studies have suggested that TP53 is upregulated in HIRI, but treatment with hydrogen-rich saline can significantly reduce TP53 expression at the mRNA and protein levels^66^. It has also been reported that NO can inhibit TP53 gene expression, thereby reducing the secretion of pro-inflammatory and chemotactic factors such as TNF-α and IL-1 to alleviate HIRI^67^. It is evident that inflammation plays a central role in the pathogenesis of HIRI.

Interestingly, EU exhibits strong anti-inflammatory effects, and previous studies have found that pretreatment with EU can improve HIRI in rats. Therefore, research focusing on inflammation may be a promising strategy for the prevention and treatment of HIRI using EU.

To explore the underlying mechanism of EU in preventing and treating HIRI, we conducted GO enrichment analysis and KEGG pathway analysis. GO enrichment analysis revealed significant enrichment in processes such as positive regulation of gene expression, positive regulation of transcription from RNA polymerase II promoter, negative regulation of apoptotic process, positive regulation of transcription, DNA-templated, and response to drug. In the top 30 enriched pathways identified by KEGG analysis, pathways involved included cancer pathway, AGE-RAGE signaling pathway in diabetic complications, lipid and atherosclerosis, TNF signaling pathway, PI3K-Akt pathway, and apoptosis. Inflammation, cell apoptosis, and oxidative stress play important roles in HIRI^60,68^. The PI3K/AKT pathway is a complex and crucial signaling pathway that dynamically regulates various processes such as inflammation, metabolism, cell apoptosis, cell survival, and cell cycle^69–71^. Various preoperative interventions act on the PI3K/AKT signaling pathway to reduce cell apoptosis, sterile inflammation, and oxidative stress, attenuating liver injury caused by transient liver ischemia and preventing the occurrence of HIRI^61–65^.

As mentioned earlier, RELA (NF-κB/p65) is a core regulatory factor of inflammatory mediators in HIRI, and mTOR activity in liver cells is dependent on the NF-κB pro-inflammatory signaling pathway mechanism to reduce the susceptibility of the liver to injury^72^. In addition, our previous research has found that chlorogenic acid can alleviate liver ischemia–reperfusion injury by suppressing HMGB1/TLR-4/NF-κB-mediated inflammation, oxidative stress, and cell apoptosis^60^. Subsequently, more studies have demonstrated the association between the mechanisms of preoperative intervention in preventing HIRI and the HMGB1/TLR-4/NF-κB-mediated inflammatory response^73–75^. Therefore, targeting the signaling pathway mediated by this pathway has great potential for the prevention and treatment of HIRI using EU.

In this study, we employed a network pharmacology approach to identify the main active ingredients and target proteins of EU in preventing and treating HIRI. We then utilized protein–protein interaction (PPI) network analysis to identify key target proteins involved in EU's effects on HIRI. Furthermore, we conducted biological enrichment analysis to explore the potential molecular mechanisms underlying EU's effects on HIRI. Our findings revealed that EU exhibits a multi-component, multi-target, and multi-pathway characteristic in its effects on HIRI, with a focus on targets and pathways associated with inflammation, oxidative stress, and cell apoptosis. We further validated our predictions through molecular docking, which showed binding energies of less than 5.0 kcal/mol, indicating that these three compounds can effectively bind to their target proteins. Among them, β-sitosterol exhibited the lowest binding energy with Akt1, suggesting a more stable interaction between β-sitosterol and Akt1 protein.

However, it is important to acknowledge some limitations of this study. Firstly, since our study relied on network databases, there may be some active ingredients and target proteins that were not included due to incomplete or unverified information in the databases. Secondly, further experimental validation is lacking for this assertion. There are research studies that have reported quercetin and naringenin were identified as the most important active ingredients of EU in preventing and treating IRI^45,76^. This research on these compounds is mostly limited to cellular and animal models. In particular, there is currently no reported research on the intervention of HIRI using β-sitosterol. Therefore, further investigation in this direction is crucial in the search for preventive drugs for HIRI, and we believe that the significance of research in this area is substantial.

## Conclusion

In this study, we employed network pharmacology methodologies to investigate the underlying mechanisms behind the protective effects of EU against HIRI. Through the screening of active compounds and target proteins, construction of protein–protein interaction networks, and biological enrichment analysis, we discovered that EU exhibits a complex profile with respect to its multi-component, multi-target, and multi-pathway characteristics in combating HIRI, primarily focusing on targets and pathways associated with inflammation, oxidative stress, and cellular apoptosis. Molecular docking results further corroborated our predictions, indicating that quercetin, β-sitosterol, and paeonol can effectively bind to their respective target proteins. Through this study, we have the opportunity to direct our future experiments, screen various drugs, and delve deep into the specific molecular mechanisms. We hold the belief that, one day in the near future, EU or one of its potent active compounds can be utilized in clinical settings to alleviate HIRI, thus eliminating its hindrance to the selection of liver transplantation for individuals with end-stage liver disease.

### Supplementary Information

Supplementary Information.

## Data Availability

All data generated or analysed during this study are included in this published article and its Supplementary information files.
