# Supplementary figures and images for: Network pharmacology and molecular docking elucidate potential mechanisms of Eucommia ulmoides in hepatic ischemia–reperfusion injury
Source: Sci Rep. 2023 Nov 24;13:20716. doi: 10.1038/s41598-023-47918-8 (PMC10673959; doi:10.1038/s41598-023-47918-8)

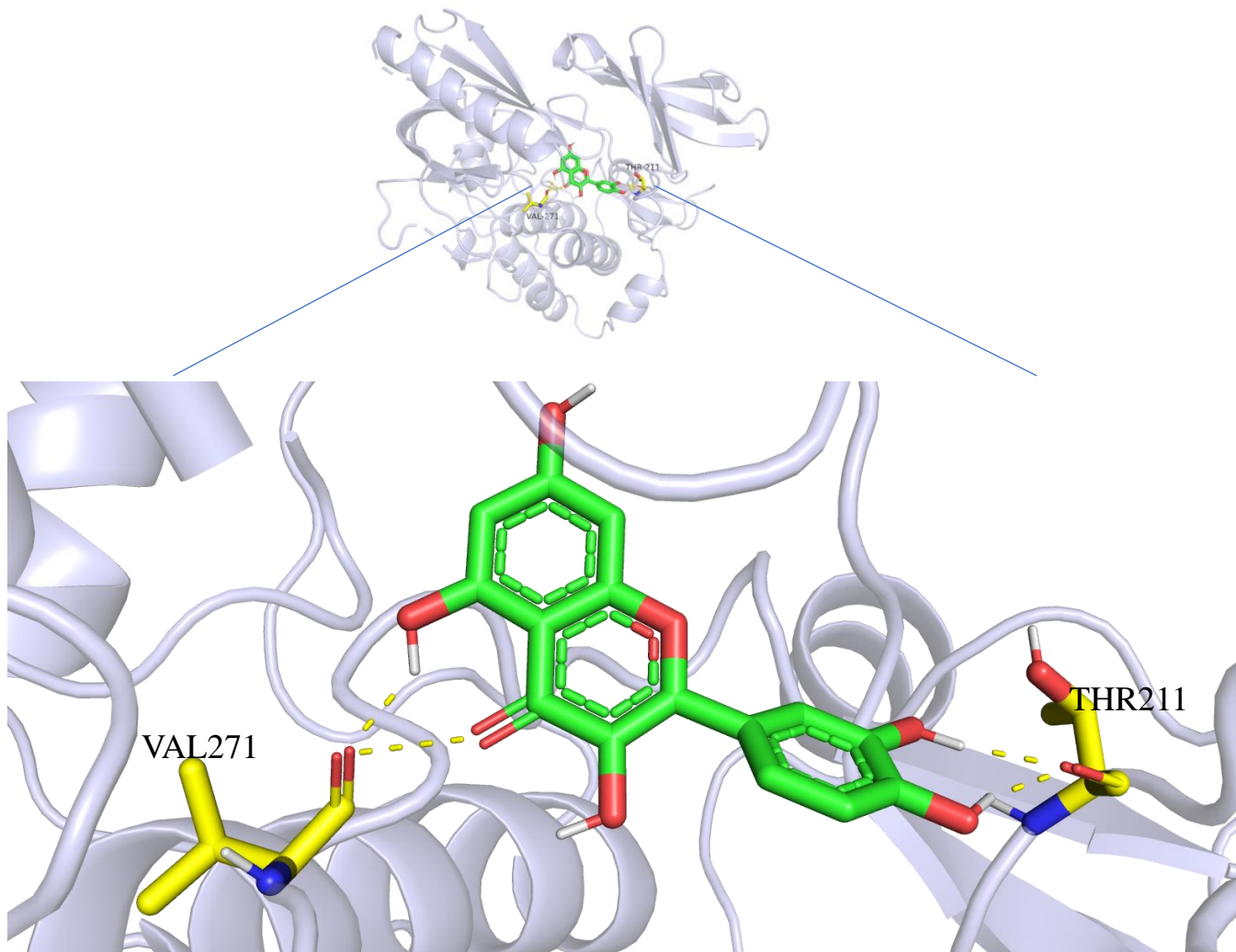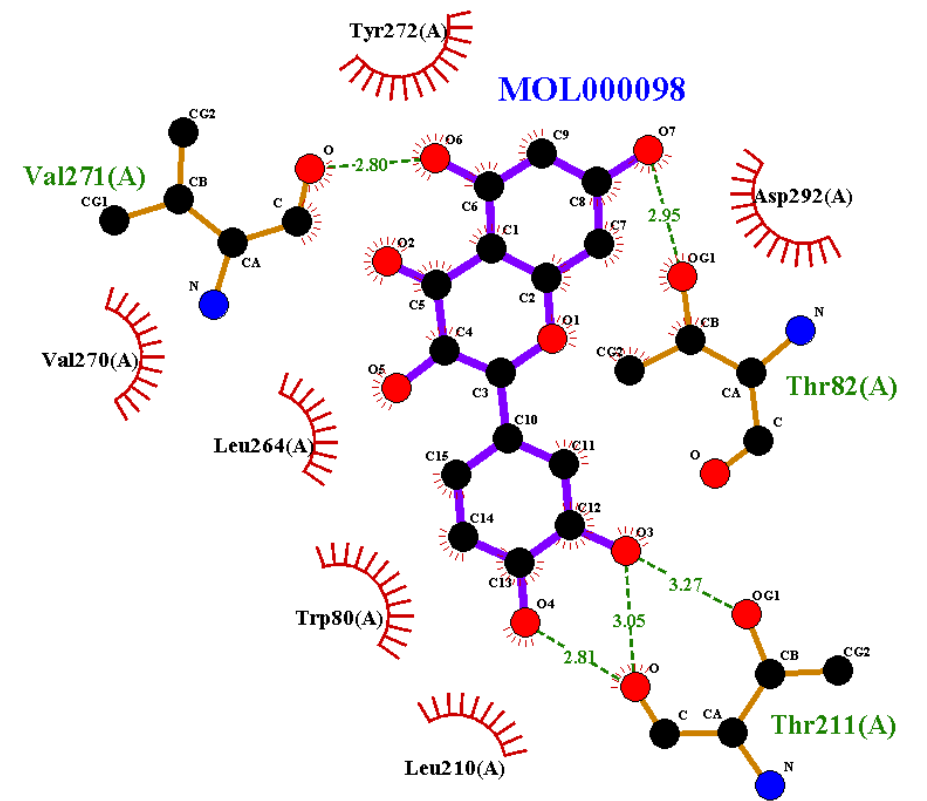

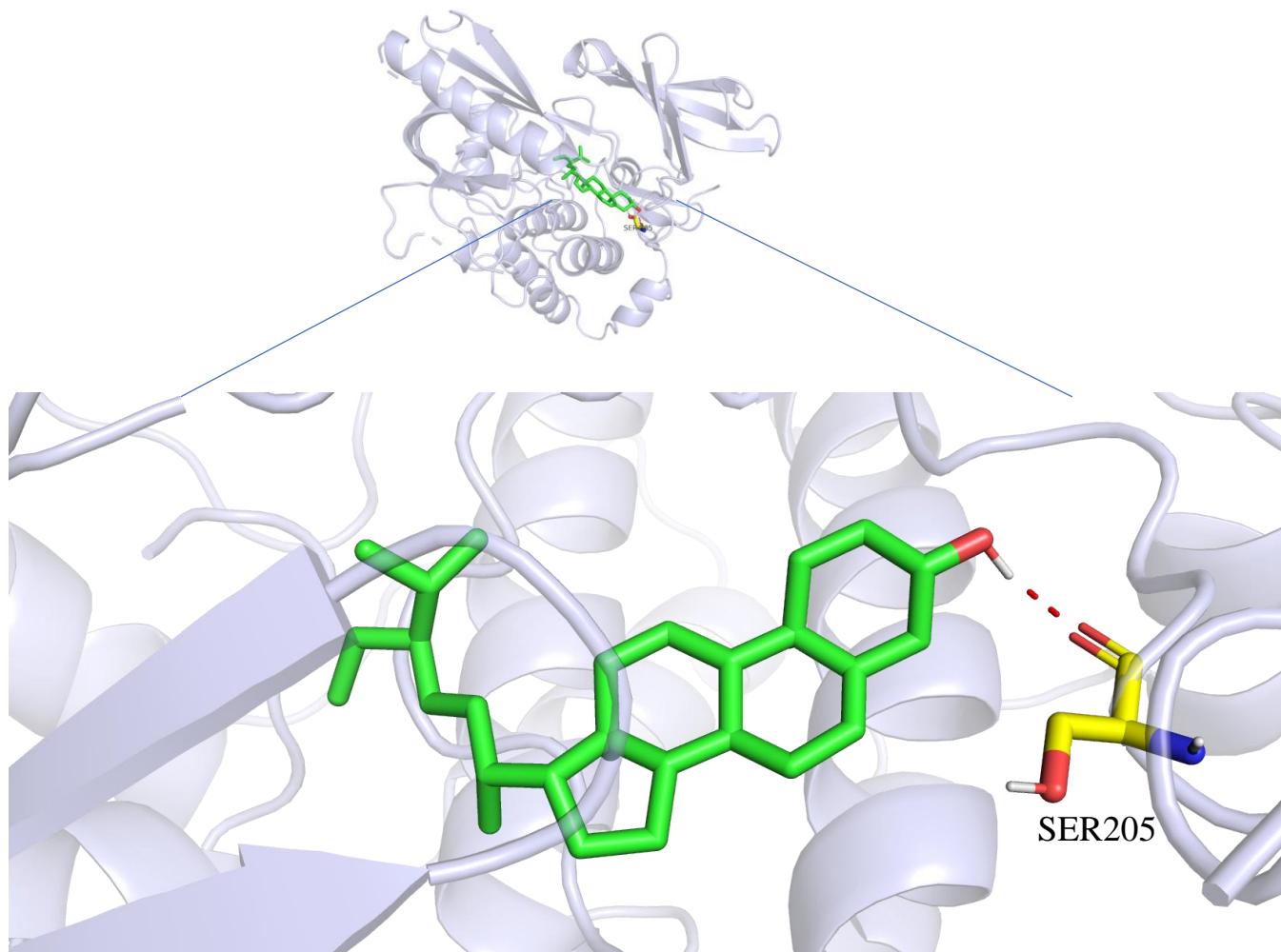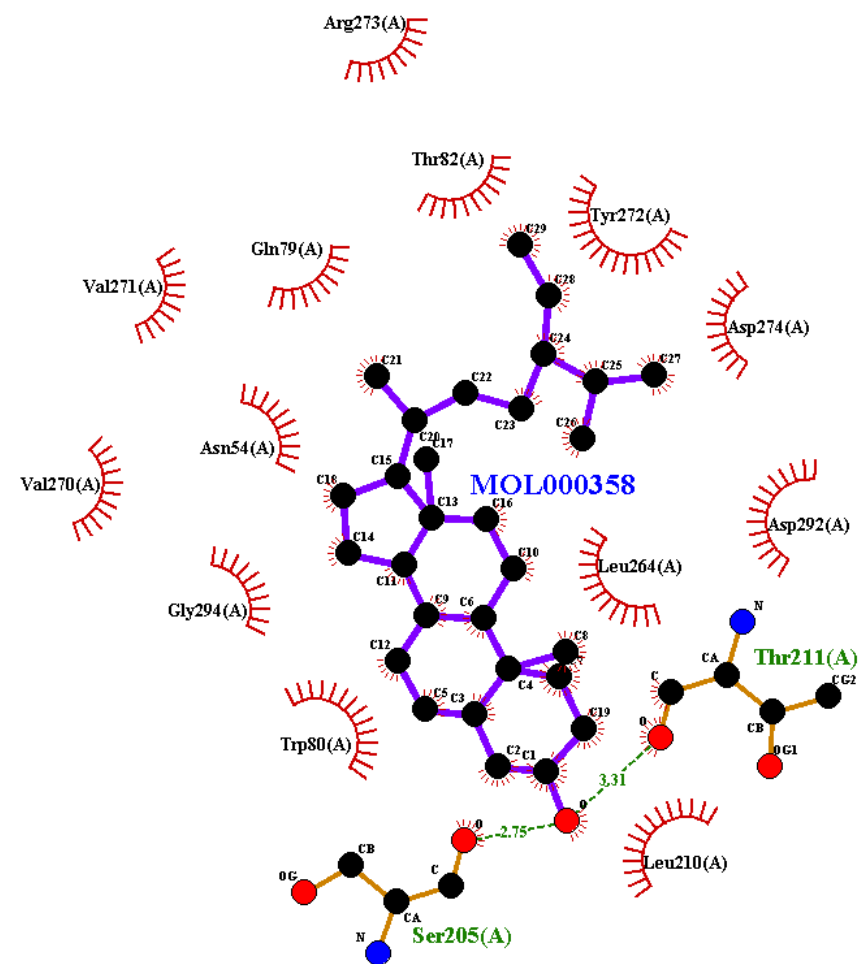

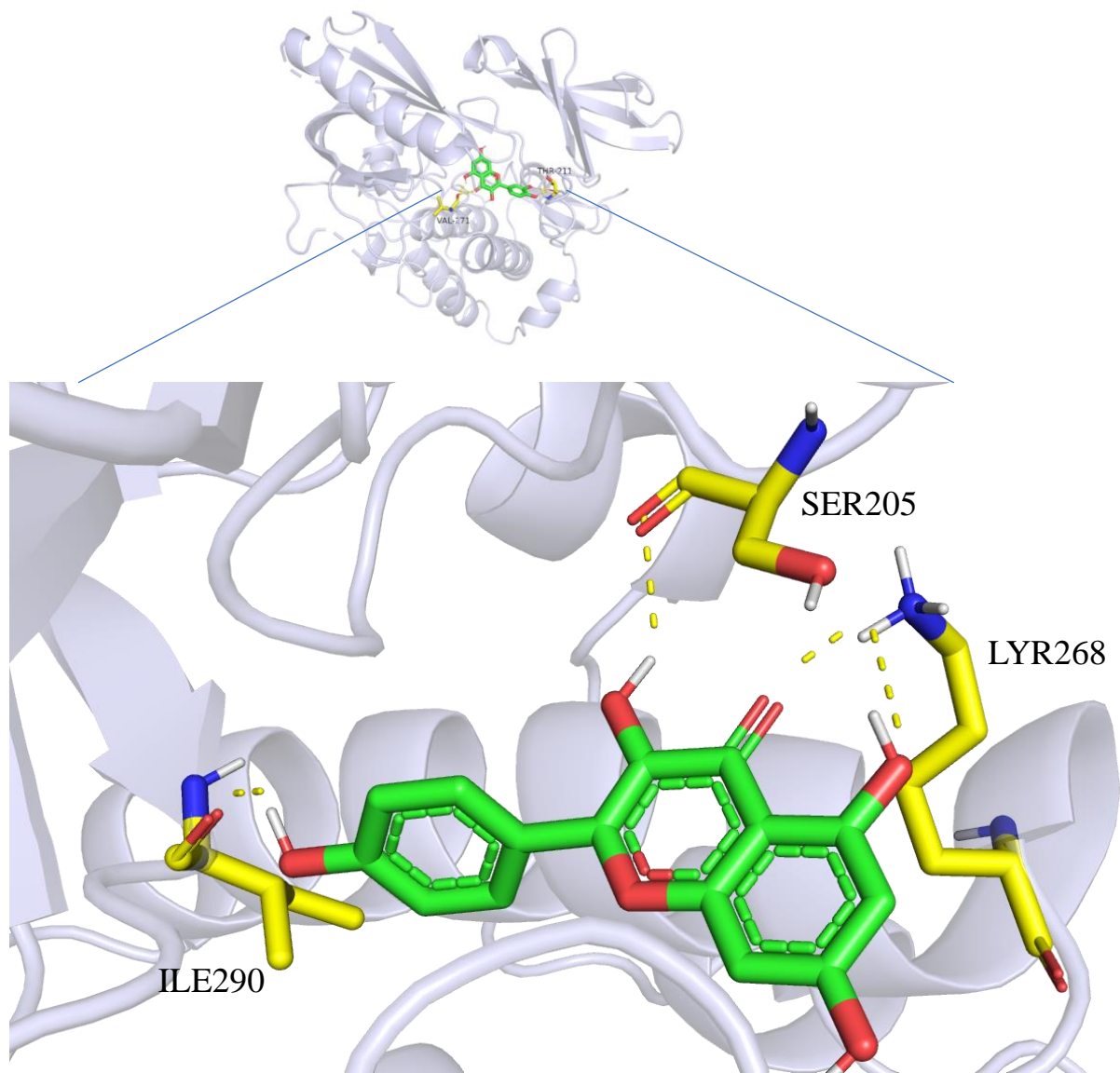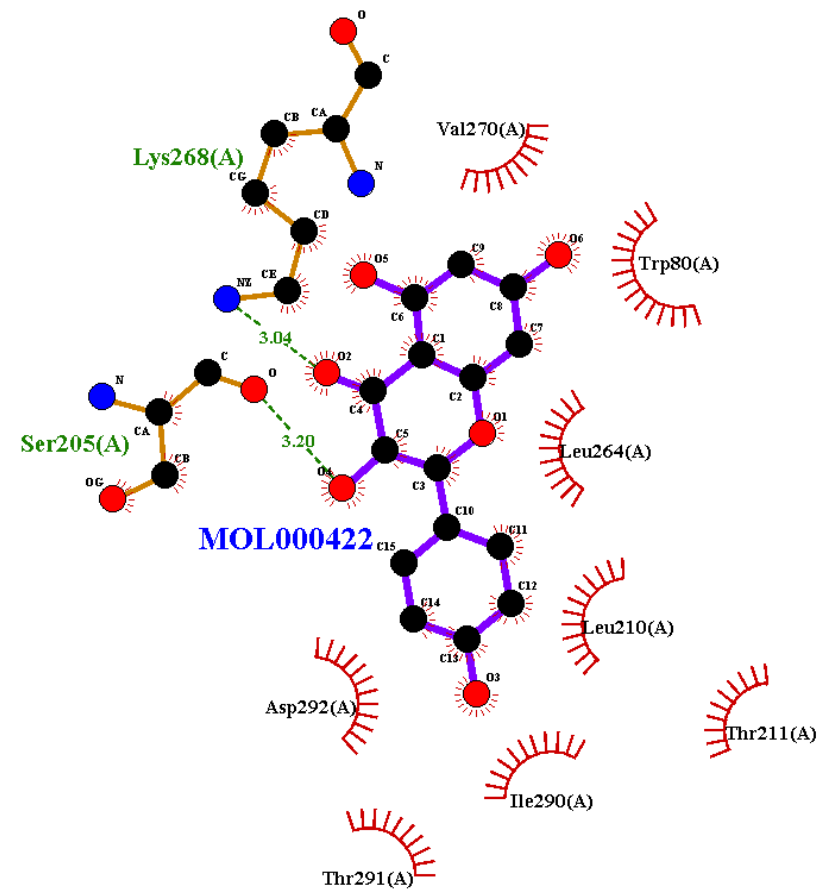

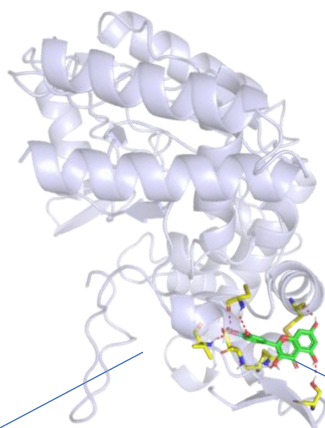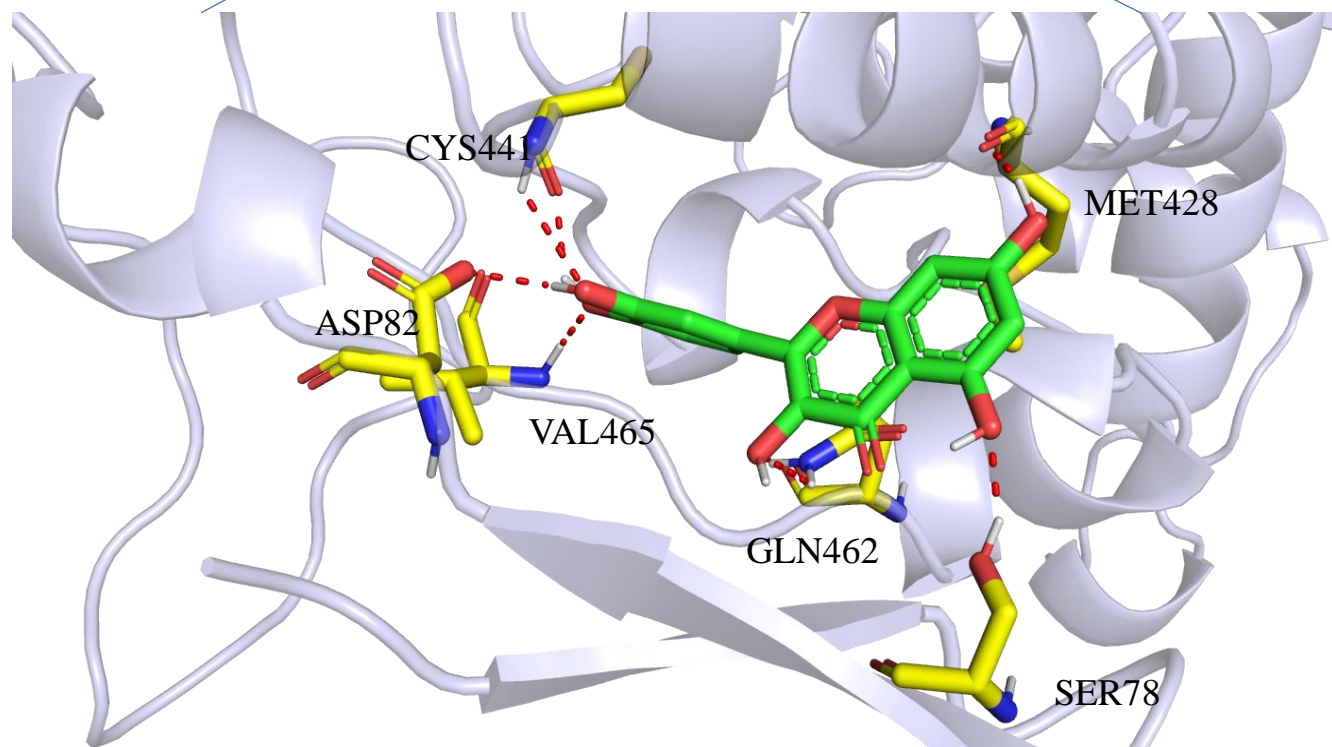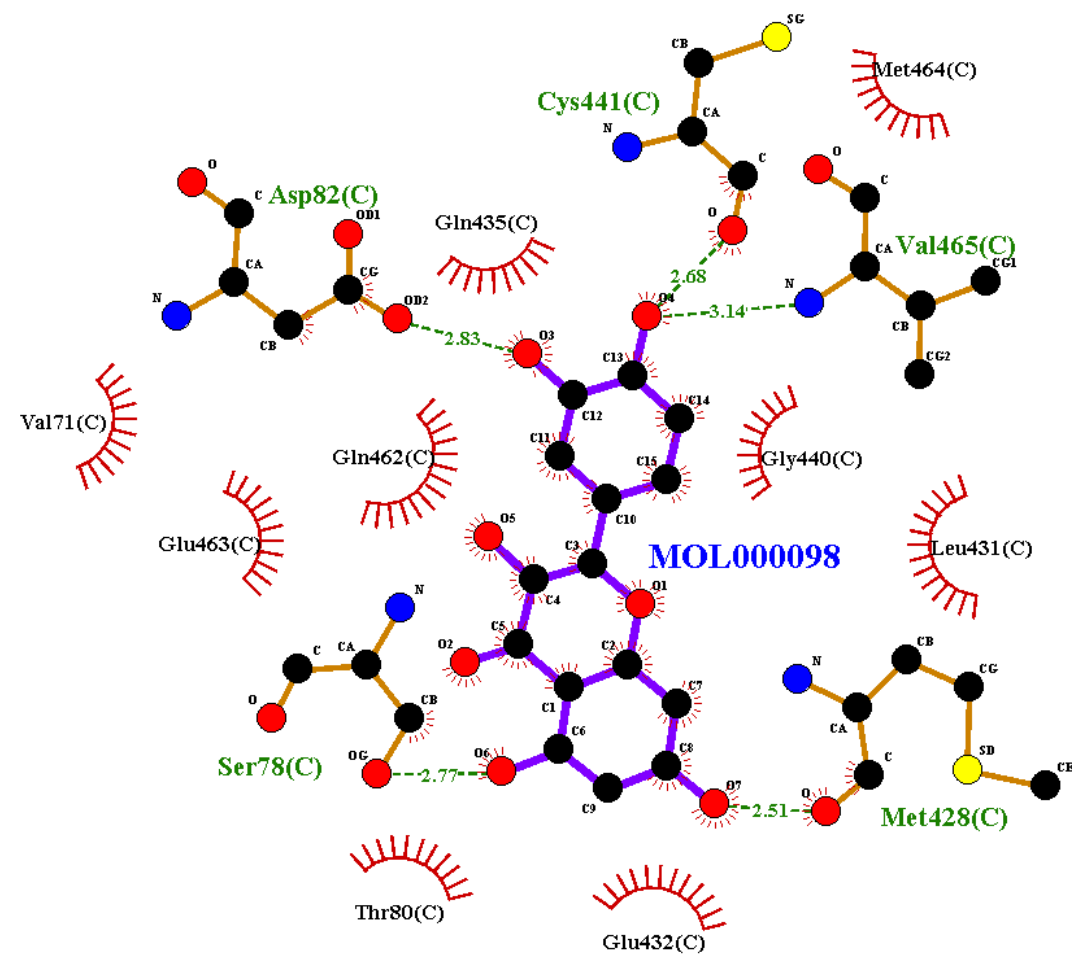

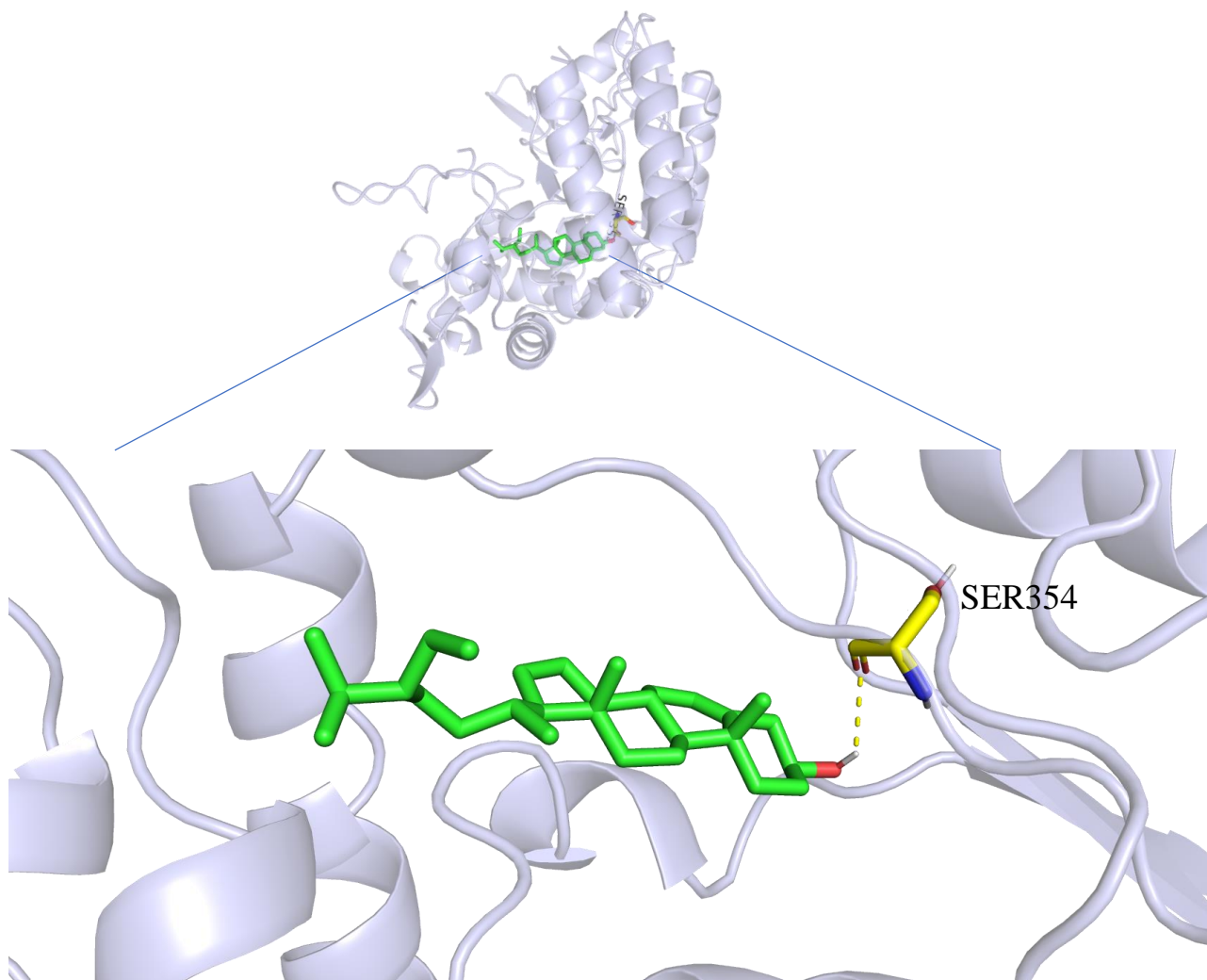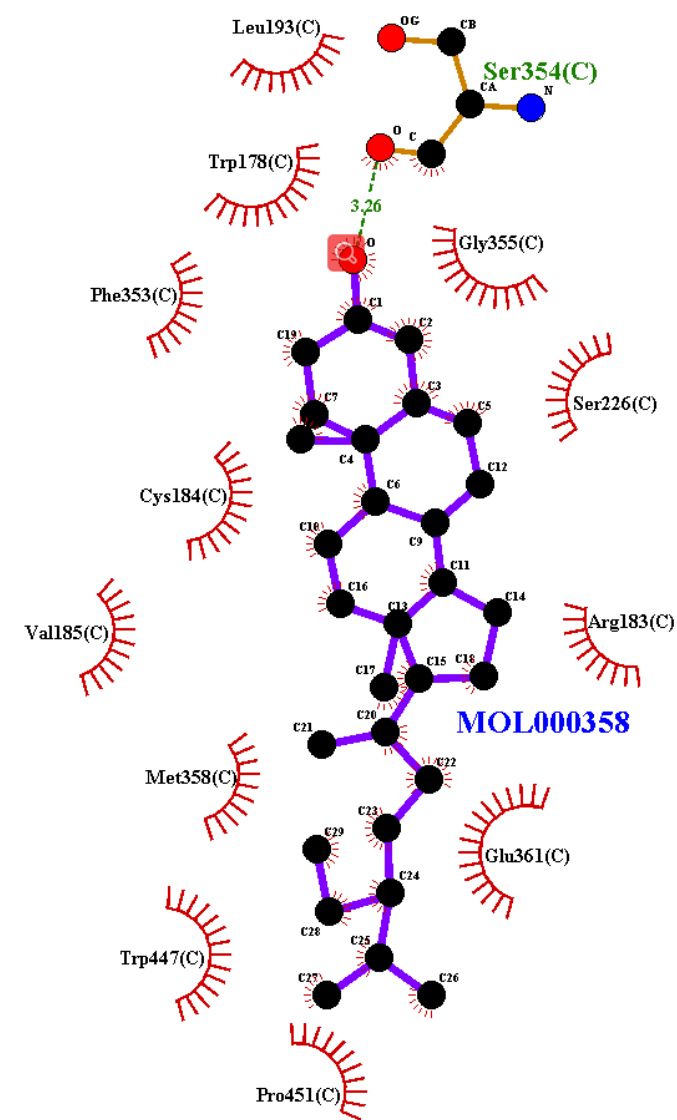

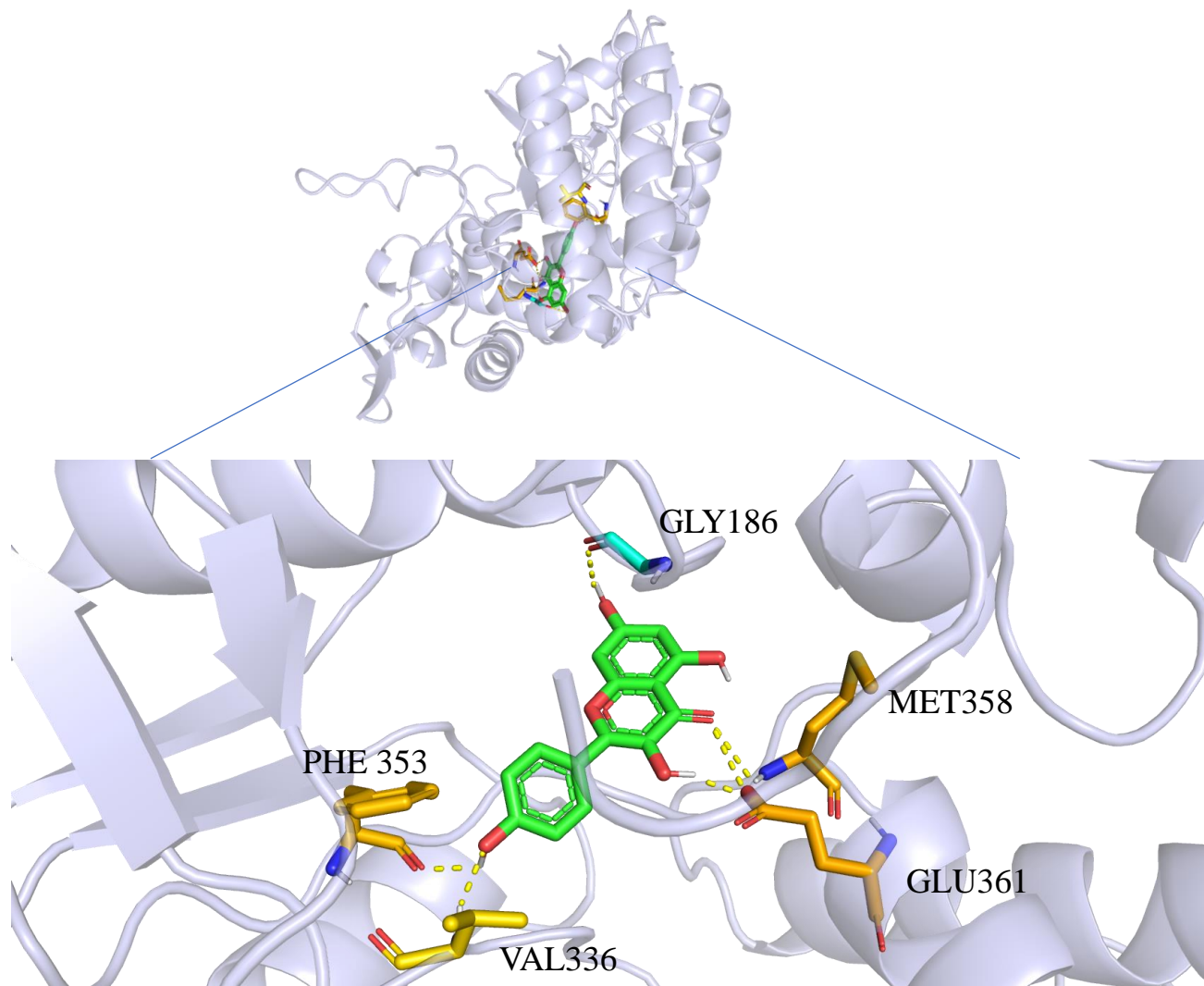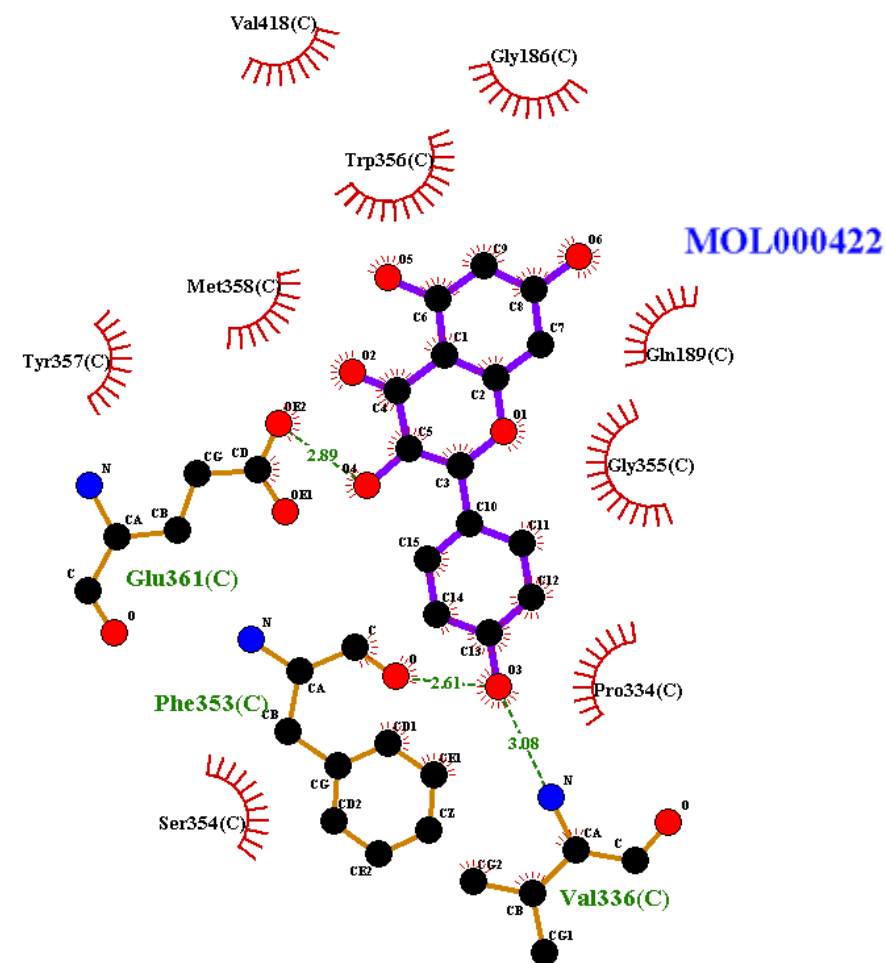

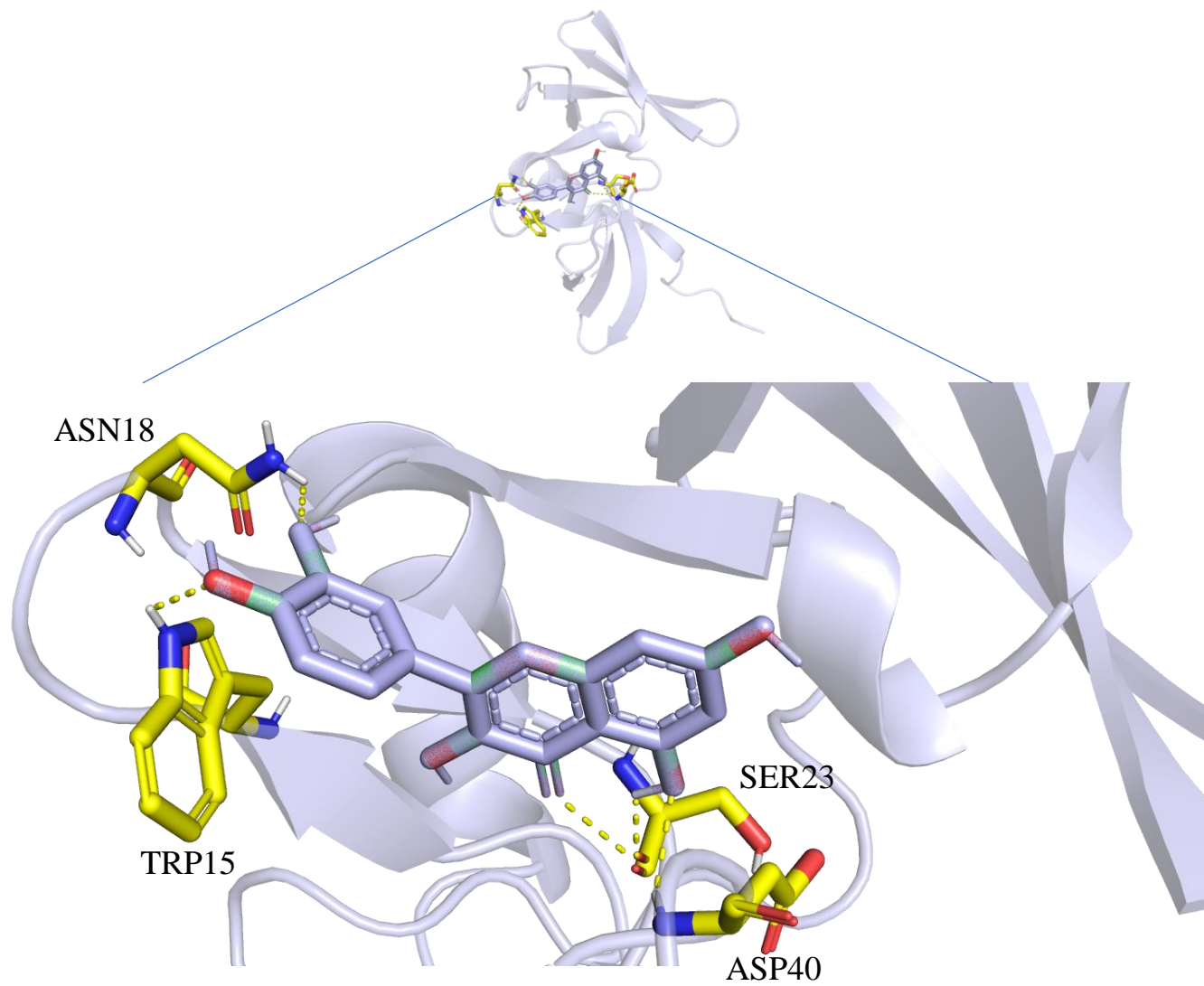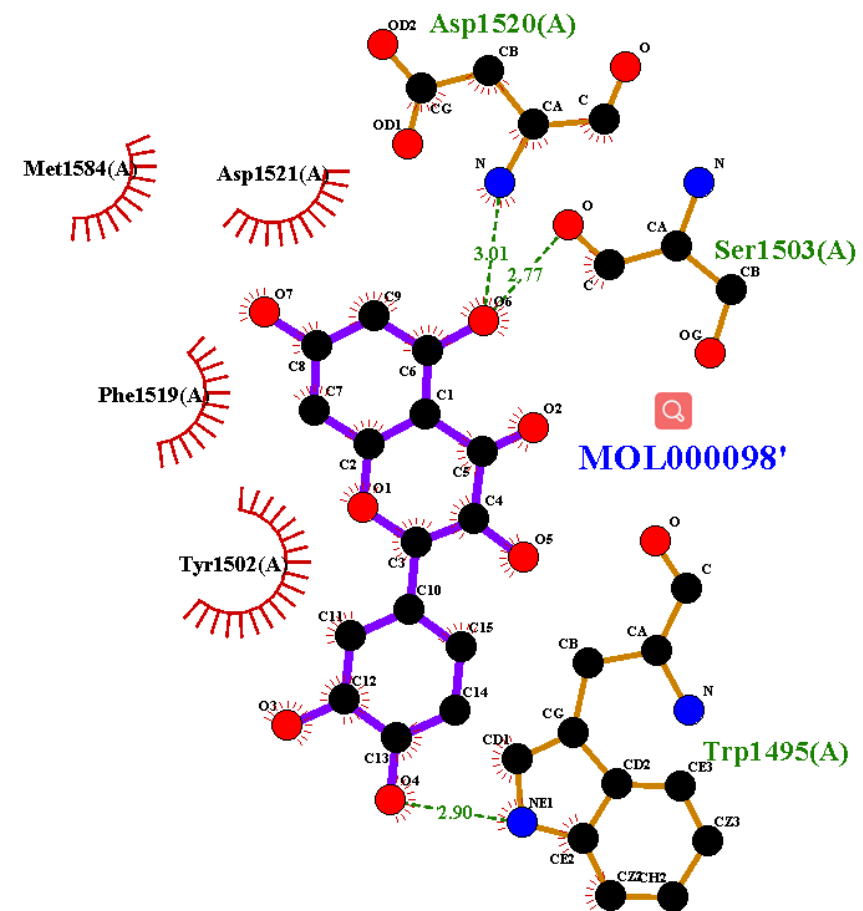

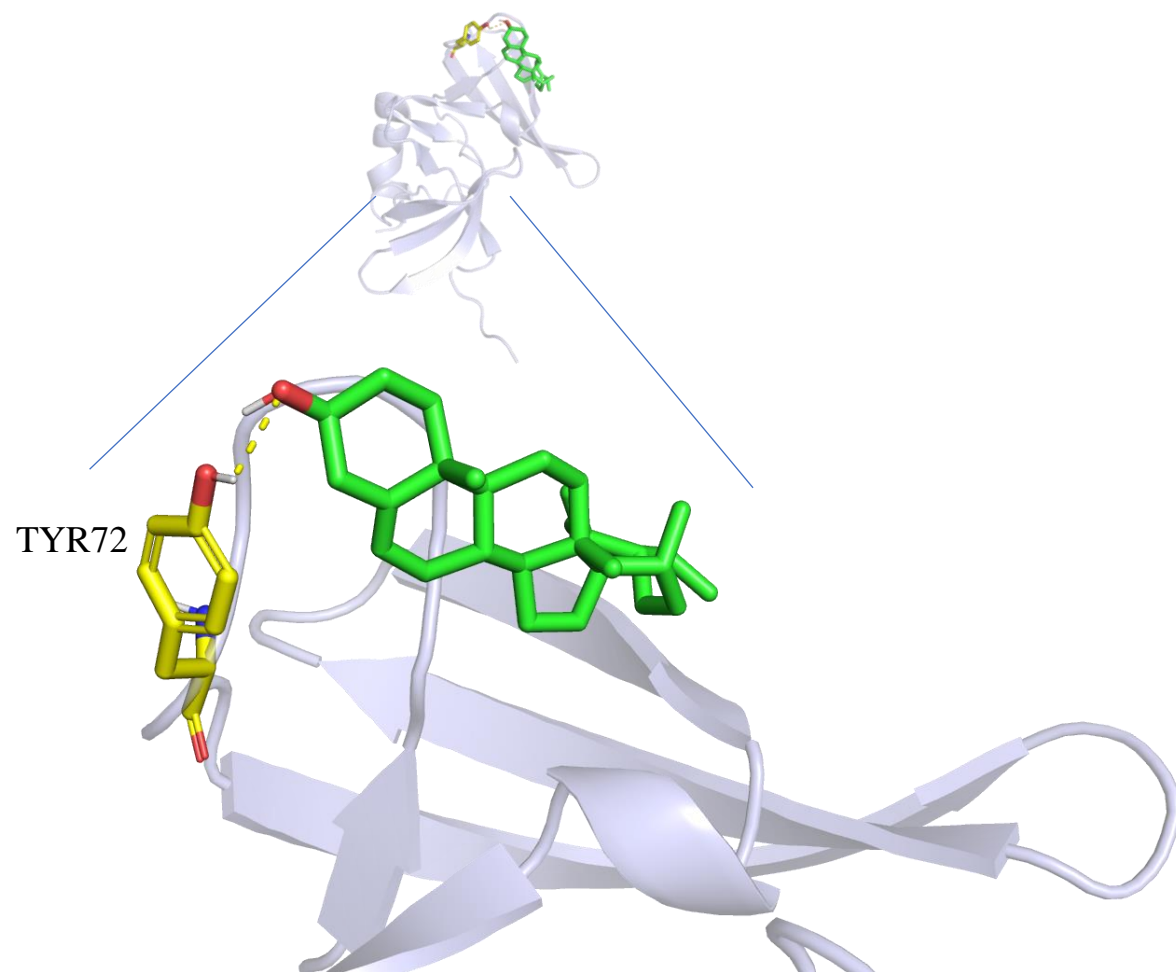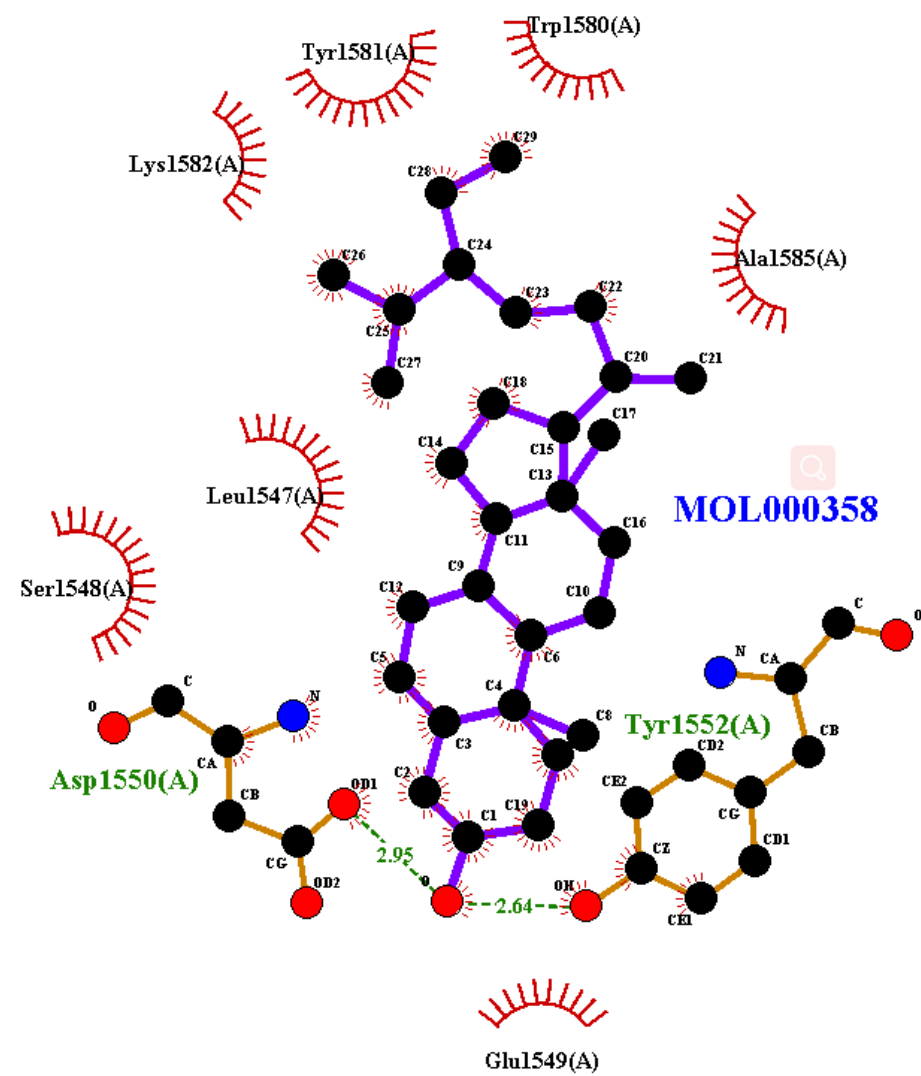

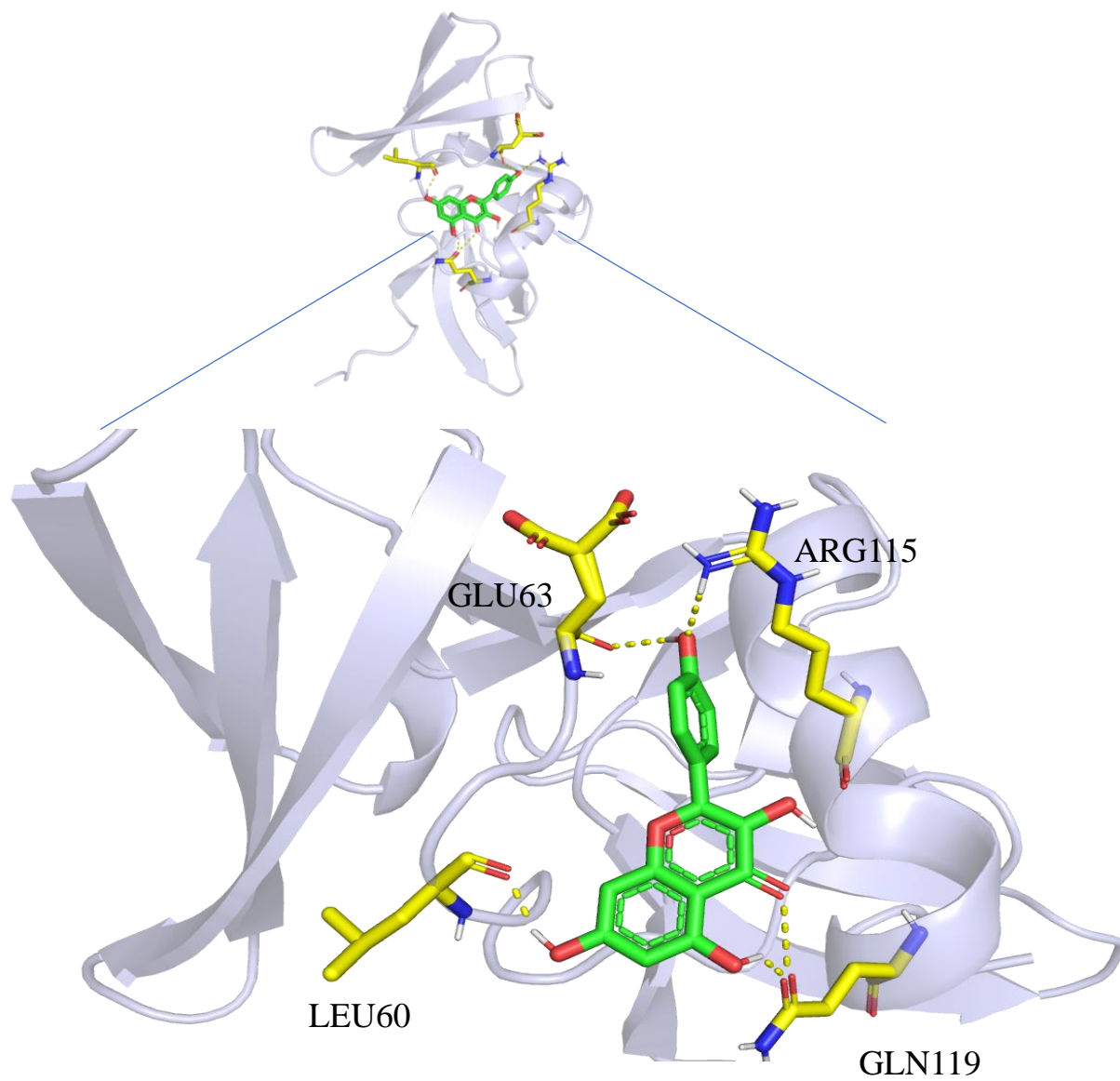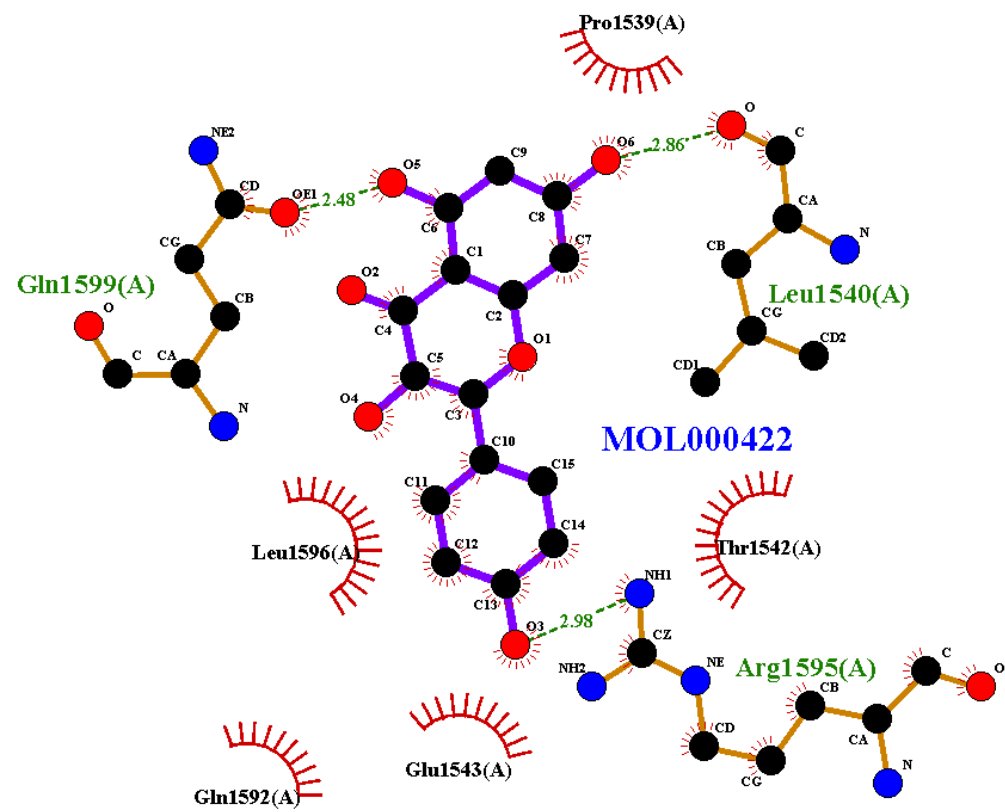

Supplement: Supplementary file 1 — Supplementary Information. [file 41598_2023_47918_MOESM1_ESM.zip › supplementary file/dock/分子对接.pdf]

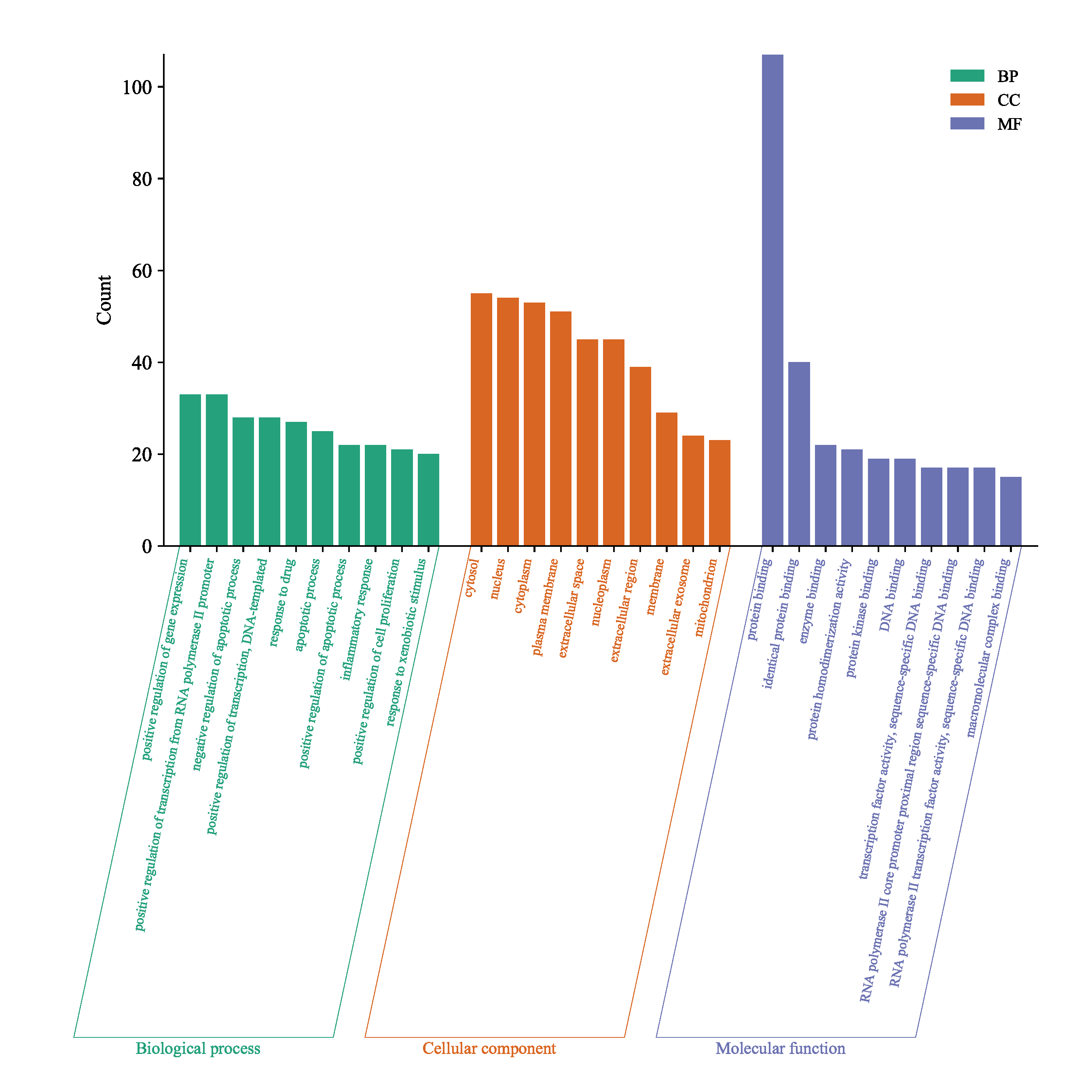

Supplement: Supplementary file 1 — Supplementary Information. [file 41598_2023_47918_MOESM1_ESM.zip › supplementary file/enrichment/Figure 4.png]

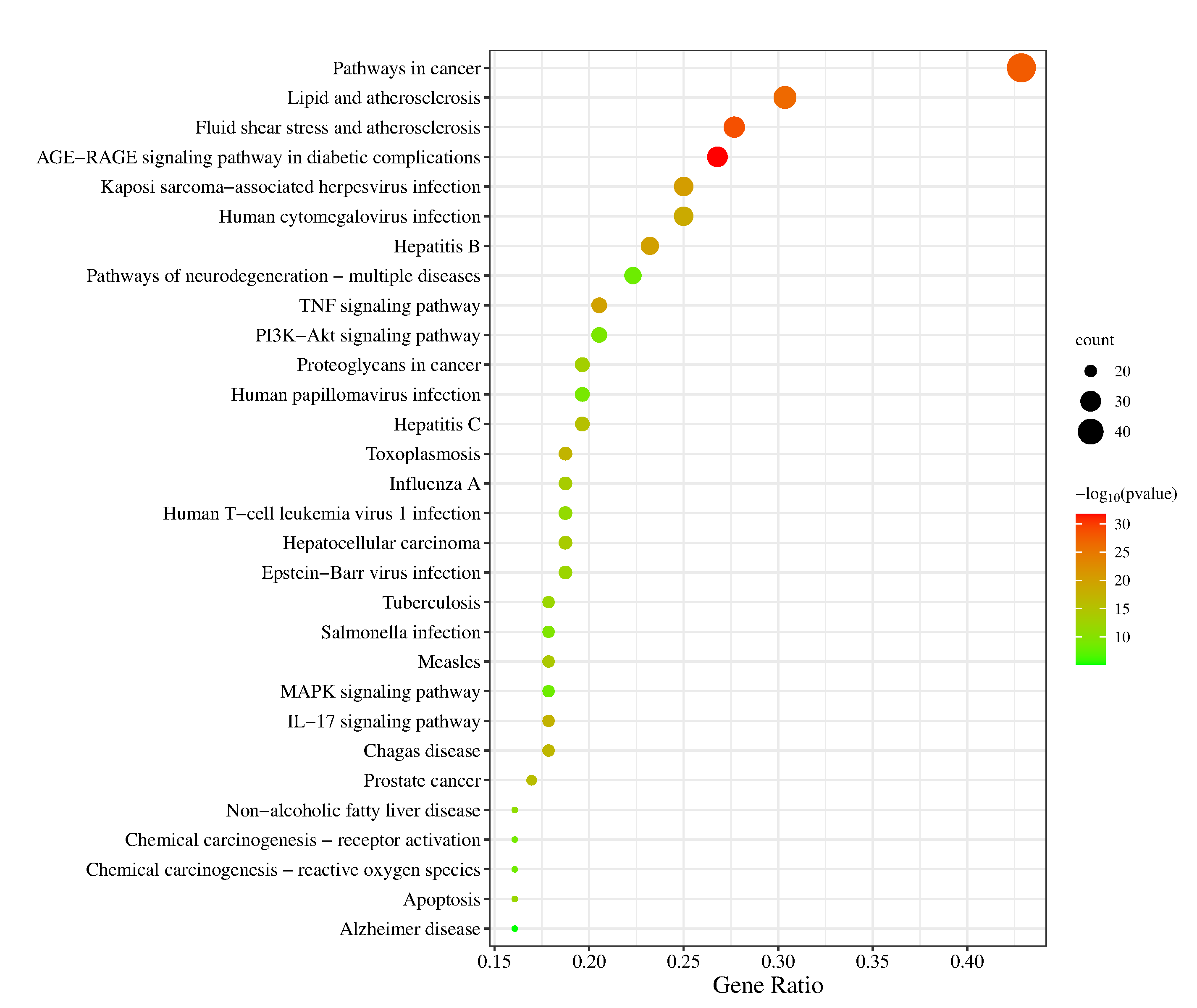

Supplement: Supplementary file 1 — Supplementary Information. [file 41598_2023_47918_MOESM1_ESM.zip › supplementary file/enrichment/Figure 5.png]

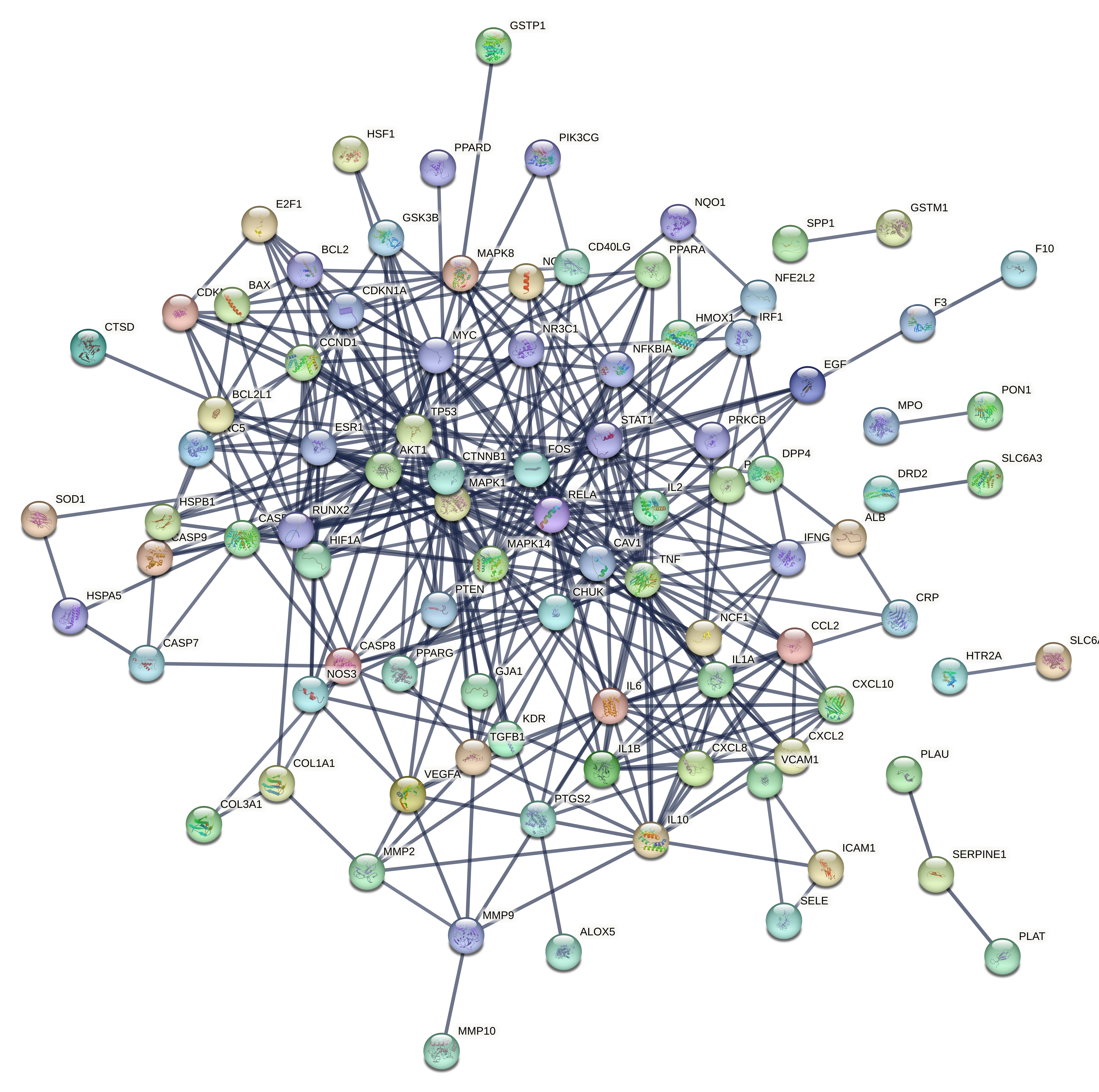

Supplement: Supplementary file 1 — Supplementary Information. [file 41598_2023_47918_MOESM1_ESM.zip › supplementary file/PPI/PPI(Fig-3B).png]
